# The deubiquitinase UCHL3 mediates p300-dependent chemokine signaling in alveolar type II cells to promote pulmonary fibrosis

**DOI:** 10.1038/s12276-023-01066-1

**Published:** 2023-08-01

**Authors:** Soo Yeon Lee, Soo-Yeon Park, Seung-Hyun Lee, Hyunsik Kim, Jae-Hwan Kwon, Jung-Yoon Yoo, Kyunggon Kim, Moo Suk Park, Chun Geun Lee, Jack A. Elias, Myung Hyun Sohn, Hyo Sup Shim, Ho-Geun Yoon

**Affiliations:** 1 Department of Biochemistry and Molecular Biology, Severance Medical Research Institute, Graduate School of Medical Science, Brain Korea 21 Project, Yonsei University College of Medicine, Seoul, 03722 Korea; 2 Department of Biomedical Laboratory Science, Yonsei University Mirae Campus, Wonju, South Korea; 3 Department of Convergence Medicine, Asan Medical Center, University of Ulsan College of Medicine, Seoul, Korea; 4 Division of Pulmonary and Critical Care Medicine, Department of Internal Medicine, Yonsei University College of Medicine, Seoul, 03722 Korea; 5 Molecular Microbiology and Immunology, Brown University, Providence, RI USA; 6 Department of Internal Medicine, Hanyang University, Seoul, 04763 Korea; 7 Department of Pediatrics and Institute of Allergy, Severance Medical Research Institute, Brain Korea 21 PLUS Project for Medical Sciences, Yonsei University College of Medicine, Seoul, 03722 Korea; 8 Department of Pathology, Yonsei University College of Medicine, Seoul, 03722 Korea

**Keywords:** Epigenetics, Cell signalling, Cancer therapy

## Abstract

Idiopathic pulmonary fibrosis (IPF) is a chronic, fatal, fibrotic, interstitial lung disease of unknown cause. Despite extensive studies, the underlying mechanisms of IPF development remain unknown. Here, we found that p300 was upregulated in multiple epithelial cells in lung samples from patients with IPF and mouse models of lung fibrosis. Lung fibrosis was significantly diminished by the alveolar type II (ATII) cell–specific deletion of the p300 gene. Moreover, we found that ubiquitin C-terminal hydrolase L3 (UCHL3)-mediated deubiquitination of p300 led to the transcriptional activation of the chemokines *Ccl2, Ccl7*, and *Ccl12* through the cooperative action of p300 and C/EBPβ, which consequently promoted M2 macrophage polarization. Selective blockade of p300 activity in ATII cells resulted in the reprogramming of M2 macrophages into antifibrotic macrophages. These findings demonstrate a pivotal role for p300 in the development of lung fibrosis and suggest that p300 could serve as a promising target for IPF treatment.

## Lung disease: role of a control protein in pulmonary fibrosis

Researchers investigating idiopathic pulmonary fibrosis (IPF), a chronic and potentially fatal lung disease of unknown cause, have identified increased levels of the epigenetic regulator protein p300 in lung cells from IPF patients and in a mouse model of the disease. The team led by Hyo Sup Shim and Ho-Geun Yoon at Yonsei University, Seoul, South Korea, also found that lung fibrosis (scar tissue formation) was significantly reduced in mice lacking the gene for p300. Chemically inhibiting p300 activity promoted the reprogramming of cells called macrophages into types able to reduce the formation of fibrosis. The research also uncovered a variety of molecular interactions involved in the initiation of fibrosis by p300, suggesting a pivotal role for the protein. Drugs targeting the production or activity of p300 might offer new approaches to treatment.

## Introduction

Idiopathic pulmonary fibrosis (IPF), one of the most common manifestations of idiopathic interstitial pneumonia, is a chronic, fatal, fibrotic, interstitial lung disease of unknown cause^1^. Many studies have attempted to elucidate the molecular mechanisms underlying pulmonary fibrosis and develop novel targeted molecular therapies^2^. The reason why IPF attracts attention is that although it is a progressive disease, there is no clear strategy to treat it. Recently, pirfenidone and nintedanib were approved as treatments for IPF; however, their effectiveness in treating fibrosis is very limited^3^. Therefore, the development of new treatments to overcome the limitations of existing treatments remains necessary^4^.

Damage to the alveolar epithelium is believed to serve as an important early pathogenic event in the development of IPF^5^. Under normal conditions, the proliferation of alveolar type II (ATII) cells and their subsequent differentiation into alveolar type I (ATI) cells contribute to alveolar repair^6,7^. However, in IPF, ATII and ATI cells fail to proliferate and are replaced by fibroblasts and myofibroblasts^8^. The loss of ATII cells damages the reparative mechanism and is thought to play a significant role in the development and progression of pulmonary fibrosis^9^. Bleomycin (BLM) increases the expression of connective tissue growth factor (CTGF), a key mediator of pulmonary fibrosis, in ATII cells, whereas CTGF blockade suppresses fibrosis development^10,11^. Additionally, a number of secreted inflammatory and profibrotic factors are released from ATII cells within the fibrotic lung^12^, suggesting that ATII cells could mediate pulmonary fibrosis in part through the secretion of profibrotic factors. Thus, a better understanding of how ATII cells function during the development of pulmonary fibrosis would provide insight into the processes associated with disease initiation and progression^13^.

Recent studies have shown that epigenetic alterations^14–16^, including histone acetylation, play pivotal roles in IPF^17–19^. For example, defective histone acetylation in the promoter of cyclooxygenase 2 (COX-2), which mediates the production of the antifibrotic factor PGE2, decreases COX-2 transcription in IPF^20^. Histone acetylation is governed by histone acetyltransferases (HATs) and histone deacetylases (HDACs)^21^. The E1A binding protein p300 (p300), which is the most widely studied HAT, regulates the transcriptional activation of various genes in response to cellular signaling pathways activated by inflammation, growth factors, and nuclear hormones^22^. Early growth response 1 (EGR1), a transcription factor activated by the transforming growth factor-beta (TGF-β) signaling pathway, induces p300 activation, which regulates the transcription of collagen genes, promoting the development of tissue fibrosis^23,24^. Recently, increased expression of active p300 was identified in fibroblasts derived from patients with IPF^25^. In addition, p300 inhibition reduces fibrotic hallmarks in both in vitro and in vivo IPF models^26^. These studies suggest that p300 in fibroblasts might serve as a therapeutic target for fibrotic diseases. Most studies on the mechanisms underlying pulmonary fibrosis, including those examining p300, have been conducted on fibroblasts, with few studies having examined the involvement of pulmonary epithelial cells, which are also believed to have a profound impact on IPF development. In addition, there have been no studies demonstrating the in vivo function of p300 in lung epithelial cells in the development of pulmonary fibrosis.

In this study, we found that the protein expression of p300 was significantly increased in lung epithelial cells, including club cells, ATII cells, and ciliated cells, in patients with IPF and mouse models of lung fibrosis. Using conditional lung epithelial cell-specific *p300* knockout mice, we demonstrated the ATII cell-specific function of p300 and the underlying mechanism contributing to the progression of pulmonary fibrosis in vivo. Collectively, our findings demonstrate the functional significance of p300 in pulmonary fibrosis and suggest that p300 could serve as a novel therapeutic target for IPF therapy.

## Materials and methods

### Patient samples

Human lung samples were obtained from the tissue bank of Severance Hospital (Seoul, Korea). This study was approved by the Ethics Committee of the Institutional Review Board of Severance Hospital (protocol no. 4.2016-0453). Tissues from patients with IPF and control samples obtained from the normal lungs of lung cancer patients were included in this study. Written informed consent was obtained from all patients. IPF patients fulfilled the diagnostic criteria established by the American Thoracic Society and the European Respiratory Society, and the diagnosis of IPF was supported by history, physical examination, pulmonary function studies, chest high-resolution computed tomography, and video-assisted thoracoscopic lung biopsy or transplant explants.

### Animal studies

All animal experiments were approved by the Institutional Animal Care and Use Committee of Yonsei University College of Medicine (Certification No. IACUC-2018-0087). The mice were housed in a specific pathogen–free animal facility with controlled temperature and humidity under a 12-h light/12-h dark cycle.

The p300 floxed mice used in this study were purchased from The Jackson Laboratory (Bar Harbor, ME, USA). To generate a conditional *p300* null allele, LoxP sites were inserted into the flanking regions of the *p300* gene on exon 9^27^. Mice with conditional *p300* deletion from ATII cells, epithelial club cells, or ciliated cells were generated by intercrossing *p300*^*f/f*^ mice with *Spc-CreER*^*T2*^, *Ccsp-CreER*^*T2*^, or *Foxj1-CreER*^*T2*^ (The Jackson Laboratory, Bar Harbor, ME, USA) mice, respectively. Before the administration of BLM, 8-week-old mice were injected with 10 mg/kg tamoxifen (Merck, Darmstadt, Germany) three times per week for 1 week. The mice were intratracheally administered PBS (vehicle control) or 4 mg/kg BLM (Santa Cruz Biotechnology, Dallas, TX, USA). An average of eight mice were used in each group.

To induce TGF-β_1_ expression in *TGF-β*_*1*_-TG mice, adult transgenic mice (8–12 weeks old) were provided drinking water containing 0.5 mg/mL doxycycline (Merck, Darmstadt, Germany) in 2% sucrose for 4 weeks. The doxycycline-containing water was replaced three times per week.

### Statistical analysis

The results were analyzed with Prism software, version 9 (GraphPad Software, San Diego, CA, USA) and are presented as the mean ± standard error of the mean (s.e.m.). Student’s *t* test was used to determine significant differences between the two groups. The Mann‒Whitney *U* test was used for post hoc analysis. When more than two groups of samples were compared, one-way ANOVA was used. Tukey’s multiple comparisons test was used for post hoc analysis of ANOVA. The significance levels are indicated as follows: n.s, not significant, *P* > 0.5; *P ≤ 0.05; **P ≤ 0.01; ***P ≤ 0.001; and ****P ≤ 0.0001.

## Results

### Alveolar type II cell-specific deletion of p300 prevents the development of lung fibrosis in mice

**Fig. 1:**
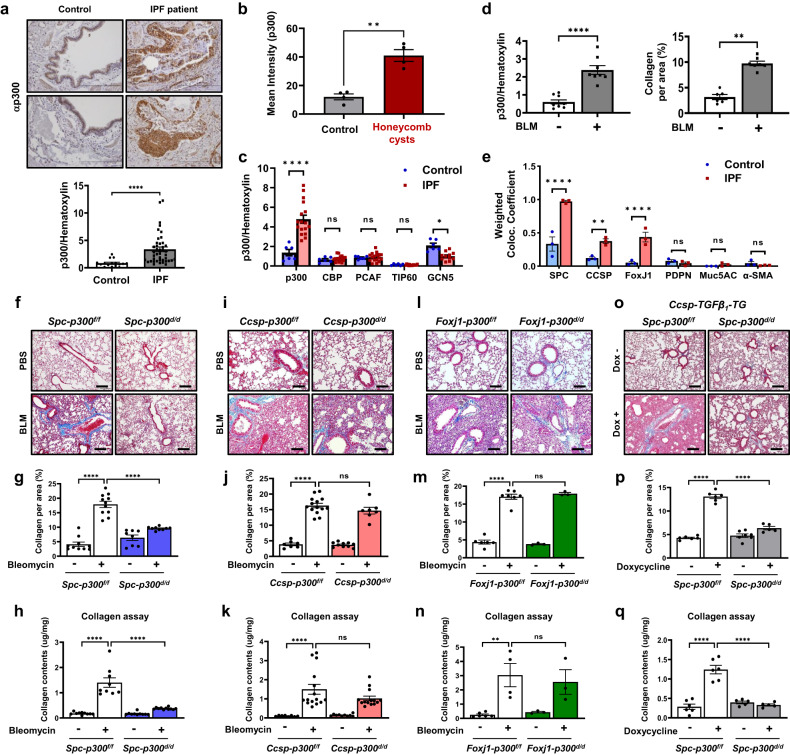
ATII cell-specific deletion of *p300* prevents lung fibrosis. **a** Representative images showing p300 IHC in lung samples from patients with IPF and control subjects. Scale bars, 100 µm. The dot plot represents the p300 intensity/hematoxylin ratio of patients with IPF (*n* = 42) and control subjects (*n* = 16). Control: 0.7293 (0.0844–2.4565) vs. IPF: 2.536 (0.6019–12.2473). **b** Immunofluorescence staining of p300 was performed on IPF lungs. The intensity of p300 was calculated using ZEN 3.0 software. *n* = 4 per group. Two-tailed *t* test with Welch’s correction. **c** The intensity of p300, CBP, PCAF, TIP60, and GCN5 protein expression was calculated using ImageJ. Control subjects (*n* = 6) and IPF (*n* = 11-17). **d** Quantitative analysis of P300 IHC and collagen in lung samples in mice with BLM-induced pulmonary fibrosis. The dot plot represents the p300 intensity/hematoxylin ratio or blue region of MTS. *n* = 5–11 mice/group. **e** Immunofluorescence analysis of human lungs using the indicated antibodies. The cell markers are as follows: Pro-SPC, ATII cells; CCSP, club cells; FoxJ1, ciliated cells; PDPN, ATI cells; Muc5AC, goblet cells; and α-SMA, fibroblasts. The weighted colocalization coefficient was calculated using ZEN 3.0 software. *n* = 3 per group. **f**, **i**, **l**, **o** Representative MTS-stained lung sections from control and cell-specific p300 KO mice ((**f**), *Spc-p300*^*f/f*^ ATII cell-specific KO; (**i**) *Ccsp-p300*^*f/f*^ club cell-specific KO; (**l**) *Foxj1-p300*^*f/f*^ ciliated cell specific; (**o**) *Spc-p300*^*f/f*^ in *Ccsp-TGF-β1*-TG mice with or without tamoxifen treatment). Scale bar, 50 µm. **g**, **j**, **m**, **p** The deposition of collagen (blue) was quantified in MTS-stained lung samples from (**g**) *Spc-p300*^*f/f*^, (**j**) *Ccsp-p300*^*f/f*^, (**m**) *Foxj1-p300*^*f/f*^, and (**p**) *Ccsp-TGF-β*_*1*_*-*TG mice using ImageJ. **h**, **k**, **n**, **q** Collagen levels in lung samples from (**h**) *Spc-p300*^*f/f*^, (**k**) *Ccsp-p300*^*f/f*^, (**n**) *Foxj1-p300*^*f/f*^, and (**q**) *Spc-p300*^*f/f*^ in *Ccsp-TGF-β*_*1*_*-*TG mice were assessed using the Sircol collagen assay. *n* = 3–15 mice/group. All average data are the mean ± s.e.m. n.s. not significant; *P < 0.05, **P < 0.01, ****P < 0.0001, Statistical analysis was performed using a two-tailed Mann–Whitney *U* test (**a**, **c**–**e**) or ANOVA with Tukey’s test (**g**–**q**).

To investigate the pathological relevance of p300 activity in pulmonary fibrosis, we first examined p300 expression in lung samples from patients with IPF and mouse models of lung fibrosis. Immunohistochemistry (IHC) showed that the lung samples of patients with IPF exhibited significantly elevated levels of p300 compared with control lung samples (Fig. 1a and Supplementary Table 1). Honeycombing is specific to pulmonary fibrosis, has a characteristic appearance of variably sized cysts, and is an important criterion in the diagnosis of IPF^1^. Thus, we next examined the expression of p300 in honeycomb cysts in IPF lung samples and normal airway regions from the control group by p300 IHC (Supplementary Fig. 1a) and immunofluorescence staining (Fig. 1b and Supplementary Fig. 1b). Increased levels of p300 were observed in the honeycomb cysts of IPF lungs compared with control lungs. The expression of p300 was highly increased in the bronchial and alveolar epithelium of IPF lungs. Notably, other HAT proteins except p300 were not increased in IPF patients compared with normal controls (Fig. 1c and Supplementary Fig. 1c).

To further verify the results obtained in lung samples from patients with IPF, we assessed changes in p300 expression in a bleomycin (BLM)-induced mouse model of lung fibrosis. An increase in p300 expression was observed in mouse lungs following BLM injection (Fig. 1d and Supplementary Fig. 2a). In addition, we examined a transgenic mouse model with inducible *TGF-β*_*1*_ overexpression (*Ccsp-TGF-β*_*1*_-TG mice), which develops lung fibrosis in response to doxycycline (Dox) administration^28^. After 28 days of Dox administration, p300 was significantly increased in the lungs of *Ccsp-TGF-β*_*1*_-TG mice compared with the lungs of control mice (Supplementary Fig. 2b).

To examine cell type–specific expression of p300 in lung samples from patients with IPF and lung fibrosis mouse models, we performed coimmunofluorescence (co-IF) staining using antibodies against pro-surfactant protein C (pro-SPC, an ATII cell marker), club cell secretory protein (CCSP, a club cell marker), forkhead box J1 (FoxJ1, a ciliated cell marker), podoplanin (PDPN, an ATI cell marker), Mucin 5AC (Muc5AC, a goblet cell marker), and α-smooth muscle actin (α-SMA, a myofibroblast cell marker). In patients with IPF, p300 expression was significantly increased in ATII cells, club cells, and ciliated cells but not in ATI cells or goblet cells (Fig. 1e and Supplementary Fig. 2c). We identified similar expression patterns in BLM-induced fibrosis model mice and *Ccsp-TGF-β*_*1*_-TG mice (Supplementary Fig. 2d). These results collectively demonstrate that p300 expression is significantly increased in the lung epithelial cells of patients with IPF and lung fibrosis mouse models.

To elucidate the physiological role of p300 in lung epithelial cells during the development of pulmonary fibrosis, we generated genetically engineered mouse models with tamoxifen-inducible *p300* knockout in ATII cells (*Spc-p300*^*d/d*^), club cells (*Ccsp-p300*^*d/d*^), or ciliated cells (*Foxj1-p300*^*d/d*^). We first verified the successful knockout of *p300* in the target lung epithelial cells of *Spc-p300*^*f/f*^, *Ccsp-p300*^*f/f*^, and *Foxj1-p300*^*f/f*^ mice by co-IF analysis using antibodies against p300 and cell type–specific markers (Supplementary Fig. 3). We next induced lung fibrosis in these model mice by BLM injection through the trachea. We found that BLM-induced lung fibrosis was markedly diminished in *Spc-p300*^*d/d*^ mice, as determined by quantifying Masson’s trichrome staining (MTS) in the lungs, soluble collagen levels, body weight, and bronchoalveolar lavage (BAL) fluid cells (Fig. 1f–h and Supplementary Fig. 4a, b). In contrast, no significant changes in fibrosis development or collagen synthesis were observed in BLM-treated *Ccsp-p300*^*d/d*^ (Fig. 1i–k and Supplementary Fig. 4c, d) or *Foxj1-p300*^*d/d*^ mice (Fig. 1l–n and Supplementary Fig. 4e, f). To further verify the ATII cell-specific role of p300 in the progression of lung fibrosis, inducible *Ccsp-TGF-β*_*1*_-TG mice were bred with *Spc-p300*^*f/f*^ mice to generate a mouse model with inducible ATII cell-specific *p300* gene deletion and *TGF-β*_*1*_ overexpression. Following doxycycline administration, control mice developed lung fibrosis; however, the development of lung fibrosis was significantly inhibited in *Spc-p300*^*d/d*^ mice (Fig. 1o–q and Supplementary Fig. 4g, h). These data suggest that p300 expression in ATII cells plays an important role in the progression of lung fibrosis.

### p300 mediates the transcriptional activation of the chemokines *Ccl2*, *Ccl7*, and *Ccl12*

**Fig. 2:**
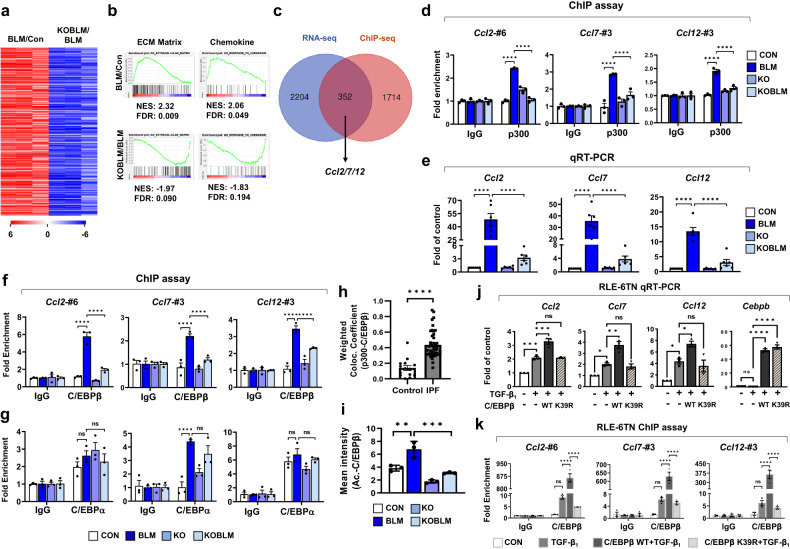
p300 regulated the chemokines Ccl2, Ccl7, and Ccl12 through C/EBPβ but not C/EBPα. **a** The heatmap represents differentially expressed gene (DEG) clusters in mouse primary ATII cells from Con and *p300* KO mice treated with or without BLM. *n* = 3 mice per group. **b** GSEA of RNA-seq signals of GO-defined ECM and chemokine gene clusters. NES normalized enrichment score, FDR false discovery rate. **c** The Venn diagram shows the overlap between differentially expressed genes and direct target genes common to the RNA-seq and ChIP-seq data. **d** ChIP assays were performed on mouse lung samples using the indicated antibodies. (*n* = 3). **e** The expression of the indicated genes in mouse lung samples was analyzed by qRT‒PCR. *n* = 6 for each group. **f**, **g** ChIP assay of C/EBPβ (**f**) and C/EBPα (**g**) binding at the p300-BE of *Ccl2*/*Ccl7*/*Ccl12* was analyzed by qPCR relative to the input DNA. *n* = 3 each. **h** Human lung samples were stained with the indicated antibodies; healthy individuals (*n* = 14), IPF patients (*n* = 44). The weighted colocalization efficiency was calculated using ZEN 3.0 software. **i** Validation of a proximity ligation assay (PLA) by visualization of the acetylated C/EBPβ protein in *Spc-p300*^*f/f*^ or *Spc-p300*^*d/d*^ mice treated with or without BLM. *n* = 3 per group. **j** RLE-6TN cells were transfected and treated with TGF-β_1_ for 24 h. *Ccl2*, *Ccl7*, *Ccl12*, and *Cebpb* gene expression was determined by qRT‒PCR. **k** RLE-6TN cells were transfected with the indicated constructs and treated with TGF-β_1_ for 6 h. A ChIP assay was performed with a C/EBPβ antibody. Statistical analysis was performed with two-way ANOVA (**d**, **f**, **g**, **k**), one-way ANOVA with Tukey’s test (**e**, **i**, **j**), or two-tailed Mann–Whitney *U* tests (**h**). Error bars represent the mean ± s.e.m. of the indicated number of independent experiments. ns not significant, *P < 0.05, **P < 0.01, ***P < 0.001 and ****P < 0.0001.

Evidence suggested that p300 mediates pulmonary fibrosis in ATII cells, and we next investigated the molecular mechanism underlying this relationship by performing RNA-sequencing (RNA-seq) analysis of murine primary ATII cells isolated from the lungs of four groups: phosphate-buffered saline (PBS)-treated *Spc-p300*^*f/f*^ mice (Con), BLM-treated *Spc-p300*^*f/f*^ mice (BLM), PBS-treated *Spc-p300*^*d/d*^ mice (KO), and BLM-treated *Spc-p300*^*d/d*^ mice (KOBLM) (Fig. 2a and Supplementary Fig. 5a). Deletion of the *p300* gene from primary ATII cells was validated by quantitative reverse-transcription–polymerase chain reaction (qRT‒PCR; Supplementary Fig. 5b). Gene set enrichment analysis (GSEA) demonstrated significant enrichment of extracellular matrix (ECM) and chemokine genes in ATII cells in the BLM group (Fig. 2b). However, p300 knockout negatively regulated BLM-induced expression of ECM and chemokine genes. The RNA-seq data identified 8,734 genes with significantly different expression levels in ATII cells in the BLM group compared with ATII cells in the Con group (fold-change > 1.5, *p* value < 0.05), including 5015 significantly upregulated genes and 3719 significantly downregulated genes. Among the upregulated genes, 2556 were downregulated when p300 was ablated in ATII cells, most of which were identified as ECM or chemokine genes (Supplementary Fig. 6a).

p300 functions as a HAT that regulates gene transcription via chromatin remodeling^29^. To identify direct target genes that are regulated by p300, we performed chromatin immunoprecipitation sequencing (ChIP-seq) of the lungs of BLM and Con mice, which identified 2,066 significantly different peaks (937 upregulated and 1129 downregulated) for p300 binding sites in fibrotic lungs compared with control lungs. Pathway analysis using EnrichR^30^ identified significant enrichment of biological processes related to macrophage and neutrophil activation and immune responses (Supplementary Fig. 6b). Comparing the ChIP-seq and RNA-seq results identified 352 genes containing p300-binding sites with altered expression patterns in ATII cells from KOBLM mice compared with BLM mice (Fig. 2c), including the chemokine genes *Ccl2*, *Ccl7*, and *Ccl12*, which were downregulated in the ATII cells of KOBLM mice and contained p300-binding elements (p300-BEs; Supplementary Fig. 6c). The transcriptional activation of *Ccl2*, *Ccl7*, and *Ccl12* in isolated primary ATII cells following BLM injection was validated by qRT‒PCR (Supplementary Fig. 6d).

To validate our ChIP-seq results, we selected putative p300-binding sites upstream of the *Ccl2* (#1–7), *Ccl7* (#1–3), and *Ccl12* (#1–3) genes (Supplementary Fig. 6c, e). The recruitment of p300 upstream of the *Ccl2*, *Ccl7*, and *Ccl12* genes in fibrotic lungs was examined by ChIP assays (Supplementary Fig. 6f-h), which revealed that p300 could bind to *Ccl2* #6, *Ccl7* #3, and *Ccl12* #3 in the context of BLM-induced lung fibrosis, and this binding was significantly decreased in the lungs of ATII cell–specific p300 knockout mice (Fig. 2d and Supplementary Fig. 6i).

To determine whether the recruitment of p300 is dependent on the p300-BE and associated with the *Ccl2*, *Ccl7*, and *Ccl12* genes, wild-type (WT) and substitution mutations (MT) of p300-BE were made on a pGL3.0-Basic plasmids containing the *Ccl2*, *Ccl7*, and *Ccl12* genes, which were then transfected into RLE-6TN ATII cells (Supplementary Fig. 7a). The ChIP assay with WT and MT p300-BE for the *Ccl2*, *Ccl7*, and *Ccl12* genes showed that the p300-BE sites were necessary for the recruitment of p300 to the *Ccl2*, *Ccl7*, and *Ccl12* genes (Supplementary Fig. 7b). In addition, mutated p300-BE in the *Ccl2*, *Ccl7*, and *Ccl12* promoters did not show TGF-β_1_-induced reporter activities (Supplementary Fig. 7c). The increased luciferase activity induced by TGF-β_1_ was significantly decreased by the knockdown or inhibition of p300, indicating that p300 regulates *Ccl2*, *Ccl7*, and *Ccl12* gene transcription by binding with p300-BE (Supplementary Fig. 7d).

We next examined changes in the expression levels of *Ccl2*, *Ccl7*, and *Ccl12* in lung samples by qRT‒PCR. Although transcriptional expression of these chemokines was elevated in the lungs of BLM mice, chemokine expression was inhibited when the *p300* gene was ablated in ATII cells (Fig. 2e). We also investigated whether p300-mediated regulation of chemokine genes was specific to ATII cells using lung epithelial, lung fibroblast, and alveolar macrophage cell lines. TGF-β_1_ treatment increased the transcription of *Ccl2*, *Ccl7*, and *Ccl12* in the RLE-6TN ATII cell line, the MLg fibroblast cell line, and the MH-S lung alveolar macrophage cell line but not in the C22 lung club cell line. However, only RLE-6TN ATII cells showed significant inhibition of the TGF-β_1_–induced increase in chemokines following *p300* inhibition (Supplementary Fig. 8a–d). We also examined the protein levels of CCL2, CCL7, and CCL12 in mouse serum and BAL fluid. As expected, the levels of all three chemokines were increased by BLM injection and were significantly decreased when *p300* was deleted from ATII cells (Supplementary Fig. 8e, f). These results suggest that p300 is selectively involved in the transcriptional regulation of the chemokines *Ccl2*, *Ccl7*, and *Ccl12* in ATII cells.

### C/EBPβ interacts with p300 to mediate the TGF-β_1_-induced transcriptional activation of chemokine genes in ATII cells

p300 is known to act as a transcriptional coactivator; therefore, motif analysis was performed to identify transcription factors involved in the transcriptional activation of *Ccl2*, *Ccl7*, and *Ccl12*. Based on the ChIP-seq results, the most commonly enriched motif identified in p300 targets was associated with the CCAAT/enhancer-binding protein (C/EBP) family, including C/EBPα and C/EBPβ (Supplementary Fig. 9a). The ChIP results showed enhanced recruitment of C/EBPβ but not C/EBPα to the p300-BE of *Ccl2*, *Ccl7*, and *Ccl12* in BLM-treated lungs, which was significantly decreased in lung samples from ATII cell–specific *p300* knockout mice (Fig. 2f, g). Moreover, knockdown of *Cebpb* but not *Cebpa* abrogated TGF-β_1_–induced transcriptional activation of *Ccl2*, *Ccl7*, and *Ccl12* in RLE-6TN cells (Supplementary Fig. 9b). *Cebpb* knockdown but not *Cebpa* knockdown reduced the promoter activity of *Ccl2*, *Ccl7*, and *Ccl12* (Supplementary Fig. 9c). TGF-β_1_ treatment significantly increased the colocalization of C/EBPβ and p300 in the RLE-6TN cell line, and this effect was decreased by treatment with the p300 inhibitor C646 (Supplementary Fig. 9d). We also observed that BLM-induced C/EBPβ and p300 colocalization was significantly decreased by *p300* knockout in ATII cells in mouse lungs (Supplementary Fig. 9e). This selective interaction between p300 and C/EBPβ was verified in BLM-treated mouse lung samples by coimmunoprecipitation (Co-IP) analysis (Supplementary Fig. 9f). We also observed that C/EBPβ and p300 colocalization in ATII cells was significantly increased in IPF lung samples compared with control lung samples (Fig. 2h and Supplementary Fig. 9g). These results suggest that p300 mediates the transcriptional activation of chemokine genes via C/EBPβ.

A previous study suggested that the acetylation of C/EBPβ K39 by p300 modulates transcriptional activity^31,32^. Therefore, we examined whether p300 acetylates C/EBPβ in fibrotic lungs to regulate the transcription of *Ccl2*, *Ccl7*, and *Ccl12*. The proximity ligation assay (PLA) results showed that C/EBPβ acetylation was significantly increased in BLM-treated mouse lung samples but not in the lungs of ATII cell–specific *p300* knockout mice (Fig. 2i and Supplementary Fig. 10a). Immunoprecipitation analysis showed that C/EBPβ acetylation was significantly increased by BLM treatment and reversed by C646 treatment (Supplementary Fig. 10b). Furthermore, TGF-β_1_–induced C/EBPβ acetylation was reduced in RLE-6TN cells following *p300* knockdown, based on PLA (Supplementary Fig. 10c) and immunoprecipitation analysis (Supplementary Fig. 10d). These data show that p300 acetylates C/EBPβ in ATII cells in response to fibrotic stimuli. We next used site-directed mutagenesis to examine whether p300-mediated acetylation of C/EBPβ K39 is required for the transcriptional activation of chemokine genes. Immunoprecipitation data showed that p300 induced less acetylation of the C/EBPβ K39R mutation than wild-type C/EBPβ (Supplementary Fig. 10e). Moreover, TGF-β_1_-induced transcriptional activation of *Ccl2*, *Ccl7*, and *Ccl12* was observed following WT C/EBPβ overexpression but not K39R mutant C/EBPβ overexpression (Fig. 2j). Finally, we observed that TGF-β_1_-induced C/EBPβ recruitment to p300-BE of the *Ccl2*, *Ccl7*, and *Ccl12* genes was reversed by the C/EBPβ K39R mutant (Fig. 2k and Supplementary Fig. 10f). These results collectively demonstrate that p300-mediated C/EBPβ acetylation is required for the TGF-β_1_–induced transcriptional activation of *Ccl2*, *Ccl7*, and *Ccl12*.

### UCHL3 deubiquitinates p300 in response to TGF-β_1_ signaling activation

**Fig. 3:**
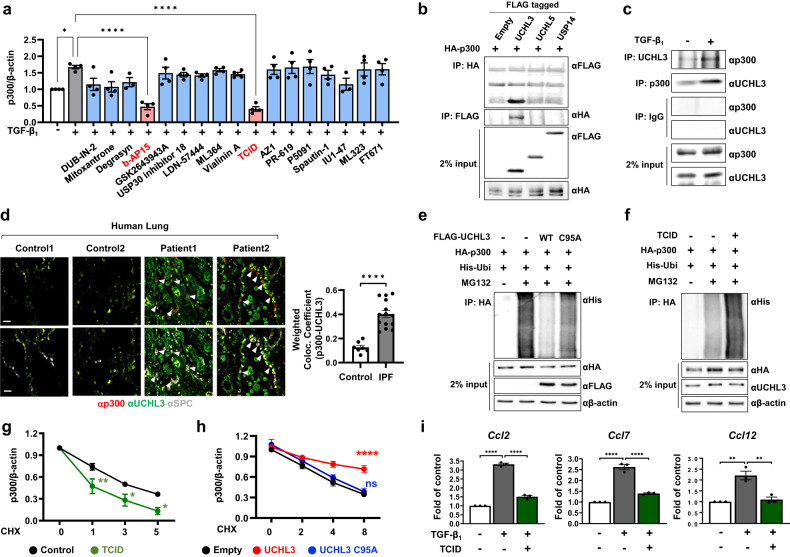
UCHL3 binds to and deubiquitinates p300 in response to TGF-β signaling. **a** Quantification of p300 protein values normalized to β-actin levels. DUB-IN-2, mitoxantrone, PR-619, and P5091 were administered at a concentration of 0.05 μM. Degrasyn, GSK2643943A, and USP30 inhibitor 18 were administered at a concentration of 0.1 μM. LDN-57444, ML364, vialinin A, and AZ1 were administered at a concentration of 1 μM. b-AP15, TCID, spautin-1, IU1-47, ML323, and FT671 were administered at a concentration of 10 μM. **b** RLE-6TN cells were transfected with the indicated constructs, and an immunoprecipitation assay was performed with FLAG or HA antibodies. **c** The lysates of RLE-6TN cells were immunoprecipitated with UCHL3 or p300 antibodies and immunoblotted with the indicated antibodies. **d** Human lung samples were stained with the indicated antibodies; healthy individuals (*n* = 7), IPF patients (*n* = 14). Arrowheads indicate colocalized regions in ATII cells. Scale bar = 50 μm. The weighted colocalization efficiency was calculated using ZEN 3.0 software. **e** RLE-6TN cells were cotransfected with the indicated plasmids. MG132 (10 μM) was added for the final 6 h. Equal amounts of protein were immunoprecipitated with HA antibodies and immunoblotted with the indicated antibodies. **f** RLE-6TN cells were cotransfected with His-ubiquitin and HA-p300 plasmids. Cells were treated 24 h posttransfection with TCID (5 μM), and whole cell lysates were prepared. **g** RLE-6TN cells were treated with TCID for 24 hr followed by treatment with 20 mg/ml cycloheximide (CHX). p300 protein levels were normalized to β-actin. **h** RLE-6TN cells were transfected with empty, UCHL3, or UCHL3^C95A^ expression vectors. Two days after transfection, the cells were treated with CHX for the indicated times. The levels of p300 protein were normalized to the level of β-actin. **i** Relative expression of *Ccl2*, *Ccl7*, *Ccl12* mRNA levels in TCID (5 µM) treated RLE-6TN. Statistical analysis was performed with one-way ANOVA with Tukey’s test (**a**,**i**), the Mann‒Whitney test (**d**) or two-way ANOVA with Sidak’s test (**g**, **h**). Error bars represent the mean ± s.e.m. ns not significant, *P < 0.05, **P < 0.01, ***P < 0.001, and ****P < 0.0001.

We found that the levels of p300 protein but not p300 mRNA were significantly increased in lung epithelial cells in lung fibrosis model mice (Supplementary Fig. 11a and 11b). Moreover, TGF-β_1_ treatment robustly increased p300 protein levels but had no effect on p300 mRNA levels in RLE-6TN cells (Supplementary Fig. 11c, d). Thus, we next investigated the molecular mechanism regulating p300 protein levels in response to TGF-β_1_ signaling activation. Protein ubiquitination is an important molecular mechanism that determines protein stability^33^, and we examined whether p300 protein levels were affected by MG132 treatment. MG132 treatment efficiently increased p300 protein levels, suggesting the involvement of ubiquitination and proteasomal degradation in the control of p300 protein stability (Supplementary Fig. 11e). Deubiquitinating enzymes (DUBs) are known to stabilize target proteins by inhibiting ubiquitin-dependent proteasomal degradation, and we hypothesized that a specific DUB was involved in the increase in p300 protein stability in response to TGF-β_1_ signaling activation. To test this hypothesis, we sought to identify the specific enzyme that changed p300 protein levels using a compound library that specifically inhibited DUBs. TCID, a selective inhibitor of ubiquitin carboxyl-terminal esterase L3 (UCHL3), robustly inhibited the increase in p300 protein levels induced by TGF-β_1_ treatment (Fig. 3a and Supplementary Fig. 12a). Notably, b-AP15, a bispecific inhibitor of UCHL5 and USP14, also significantly reduced p300 protein levels. However, co-IP analysis showed that p300 could bind to UCHL3 but not UCHL5 or USP14, indicating that UCHL3 specifically binds to and stabilizes p300 in the context of TGF-β_1_ signaling (Fig. 3b). Mapping analysis showed that UCHL3 directly interacted with the bromodomain of p300 (Supplementary Fig. 12b–d). The interaction between UCHL3 and p300 was significantly increased by TGF treatment (Fig. 3c). TGF-β_1_ treatment significantly increased the colocalization of UCHL3 and p300 in the RLE-6TN cell line, and this effect was decreased by treatment with TCID (Supplementary Fig. 13a). We also verified that p300 and UCHL3 colocalization in ATII cells was significantly increased in IPF lung samples compared with control lung samples (Fig. 3d). Importantly, wild-type UCHL3 but not inactive mutant UCHL3^C95A^ efficiently reduced p300 ubiquitination (Fig. 3e). In addition, TCID treatment significantly enhanced the ubiquitination of p300 (Fig. 3f). We also observed that the half-life of p300 in cells treated with TCID was significantly shorter than that in control cells (Fig. 3g and Supplementary Fig. 13b). As expected, overexpression of wild-type UCHL3 increased the half-life of p300 compared with that of the inactive mutant UCHL3^C95A^ (Fig. 3h and Supplementary Fig. 13c). Consequently, TGF-β_1_-induced transcriptional activation of *Ccl2*, *Ccl7*, and *Ccl12* was significantly decreased in RLE-6TN cells treated with TCID (Fig. 3i). Furthermore, TCID treatment dramatically abolished the interaction and colocalization of C/EBPβ and p300 in the RLE-6TN cell line (Supplementary Fig. 13d, e). These results collectively demonstrate that UCHL3 directly deubiquitinates and stabilizes p300 and mediates p300-dependent transcriptional activation of chemokine genes via C/EBPβ.

### p300 selectively mediates chemokine secretion to promote macrophage polarization in ATII cells

**Fig. 4:**
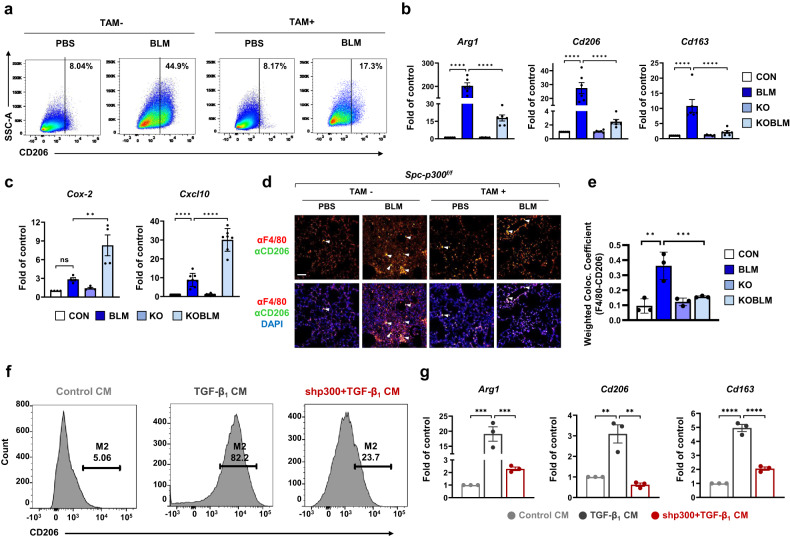
ATII cell–specific deletion of p300 inhibits macrophage polarization. **a** Flow cytometry showed the percentages of M2 (CD45^+^F4/80^+^CD206^+^) macrophages in the tissues of *Spc-p300*^*f/f*^ or *Spc-p300*^*d/d*^ mice. SSC, side scatter. **b**, **c** Relative expression of *Arg1*, *Cd206, Cd163, Cox-2, and Cxcl10* in mouse lung samples. *n* = 6. **d**, **e** Immunofluorescence analysis of CD206 (green) and F4/80 (red) with DAPI (blue) in the lungs. Arrowheads indicate colocalized cells. Scale bar = 50 μm. **f** Flow cytometric analysis of the expression of the M2 marker CD206 in MH-S murine alveolar macrophages cultured with or without CM from control or p300 knockdown RLE-6TN cells. **g** qRT‒PCR analysis of the M2 markers *Arg1*, *Cd206*, and *CD163* in MH-S cells cultured with or without CM from control or p300 knockout RLE-6TN cells. Statistical analysis was performed with ANOVA with Tukey’s test. Error bars represent the mean ± s.e.m. ns not significant, **P < 0.01, ***P < 0.001, and ****P < 0.0001.

Chemokines, such as CCL2, CCL7, and CCL12, regulate macrophage polarization under fibrotic conditions^34^. We examined whether p300 mediates pulmonary fibrosis by promoting macrophage polarization. Pulmonary macrophages from lung samples and BAL fluid were analyzed by flow cytometry using CD45^+^F4/80^+^CD206^+^ marker expression to identify M2 macrophages (Supplementary Fig. 14a). M2 macrophages were increased in lung samples and BAL fluid following BLM injection but were significantly decreased following p300 knockout in ATII cells (Fig. 4a and Supplementary Fig. 14b–d). As shown in Fig. 4b, qRT‒PCR analysis of M2 macrophage markers in the lungs revealed that the expression of *Arg1*, *Cd206*, and *Cd163* was elevated in BLM-induced mice relative to control mice. Parallel studies demonstrated that these genes were significantly downregulated following *p300* knockout in ATII cells, yielding levels of M2 macrophage markers similar to those observed in control mice. Moreover, the mRNA levels of the antifibrotic markers *Cox-2* and *Cxcl10* were significantly elevated by *p300* knockout in ATII cells in BLM-treated mouse lungs (Fig. 4c). IF staining of an M2 macrophage marker (CD206) further verified that CD206-positive macrophages were abundant in fibrotic lungs but were strongly reduced in ATII cell–specific *p300* knockout lungs (Fig. 4d, e). We next examined whether chemokines produced by ATII cells affected macrophage polarization by treating alveolar macrophages with conditioned media (CM). We found that TGF-β_1_-treated CM strongly induced the expression of M2 macrophage markers, whereas *p300* knockdown CM suppressed the induction of M2 macrophage markers, as shown by qRT‒PCR analysis of *Arg1* and *Cd163*, as well as qRT‒PCR and flow cytometric analysis of CD206 (Fig. 4f, g, and Supplementary Fig. 14e). These data demonstrate that p300 mediates M2 macrophage polarization to promote pulmonary fibrosis in an ATII cell-specific manner.

### Selective blockade of p300 activity or stability suppresses pulmonary fibrosis by reprogramming M2-like macrophages into antifibrotic macrophages

**Fig. 5:**
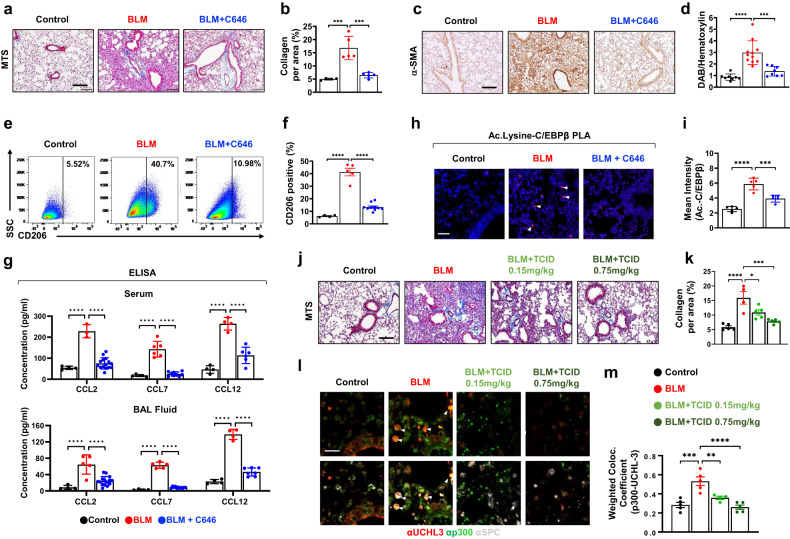
Selective inhibition of p300 prevents the progression of lung fibrosis by suppressing the production of chemokines and macrophage polarization. **a** Representative MTS-stained lung sections from BLM-treated mice treated with control or C646. **b** The deposition of collagen was quantified in MTS-stained lung samples from BLM-treated mice treated with control or C646. *n* = 4–5 per group. **c** Immunohistochemical images of α-SMA protein in lung samples from BLM-treated mice treated with control or C646. **d** The α-SMA intensity/hematoxylin ratio was quantified using ImageJ software. *n* = 8–11 per group. **e** Flow cytometric analysis of M2 (CD45^+^/F4/80^+^/CD206^+^) cells in the lungs. *n* = 4–5 per group. **f** Quantification of the percentage of M2 macrophages. **g** ELISA analysis of CCL2, CCL7, and CCL12 protein levels in serum and BAL fluid from control or C646-treated mice. **h** PLA images using Ac.-lysine and C/EBPβ antibodies to analyze lung samples from BLM-treated with control or C646. Arrowheads indicate positive signals. Scale bar, 10 μm. **i** The mean intensity of the PLA-positive signal was quantified using ZEN 3.0 software. *n* = 5 per group. **j** Representative MTS-stained lung sections from BLM-treated mice treated with control or TCID (0.15 or 0.75 mg/kg). **k** Collagen deposition was quantified in MTS-stained lung samples from BLM-treated mice treated with control or TCID. *n* = 3–5 per group. **l** Colocalization of p300 (green), UCHL3 (red), and SPC (gray) by IF staining in the indicated mouse lung samples. Scale bar, 50 μm. *n* = 3. **m** The graph shows the weighted colocalized coefficiency. Error bars, mean ± s.e.m. **P < 0.01, ***P < 0.001 and ****P < 0.0001, one-way ANOVA followed by Tukey’s test.

Our findings suggested that p300 acts as a key mediator of pulmonary fibrosis; therefore, we tested whether the selective inhibition of p300 suppressed pulmonary fibrosis by suppressing M2 macrophage polarization. BLM-treated mice were intraperitoneally injected every other day beginning on Day 1 with vehicle or C646, a selective p300 inhibitor (Supplementary Fig. 15a). The mice were sacrificed on Day 14, and BAL fluid and blood were immediately collected, followed by lung resection for α-SMA IHC and the quantification of soluble collagen levels. BLM-treated mice that were injected with C646 showed no evidence of collagen deposition, as determined by MTS analysis of lung sections (Fig. 5a, b). Moreover, C646 treatment reduced the expression of α-SMA, a marker of activated myofibroblasts (Fig. 5c, d). M2 macrophage polarization induced by BLM was efficiently inhibited by C646 treatment in lung samples and BAL cells (Fig. 5e, f, and Supplementary Fig. 15b, c). As shown in Supplementary Fig. 15d, the mRNA levels of M2 macrophage markers were significantly decreased in lung samples following C646 treatment, and C646 treatment could increase the mRNA levels of the antifibrotic markers *Cox-2* and *Cxcl10*. As expected, enhanced mRNA and protein expression levels of CCL2, CCL7, and CCL12 were induced by BLM treatment and reversed by C646 treatment (Fig. 5g and Supplementary Fig. 15e). Consistent with these data, the increased recruitment of p300 and C/EBPβ but not C/EBPα to the p300-BE regions of *Ccl2*, *Ccl7*, and *Ccl12* following fibrotic stimuli was significantly decreased in the lungs of C646-treated mice (Supplementary Fig. 15f). Additionally, we confirmed that TGF-β_1_-induced C/EBPβ recruitment was inhibited by C646 treatment in the RLE-6TN cell line (Supplementary Fig. 15g). PLA and IF analysis showed that the acetylation of C/EBPβ and the colocalization of p300 and C/EBPβ in ATII cells were increased in BLM-treated mouse lungs but were significantly decreased in C646-treated lungs (Fig. 5h, i, and Supplementary Fig. 15h). These data suggest that the selective inhibition of p300 abrogates pulmonary fibrosis by suppressing ATII cell–dependent chemotactic signaling.

We next examined whether selective inhibition of UCLH3 alleviated pulmonary fibrosis by suppressing p300/C/EBPβ-mediated chemokine signaling (Supplementary Fig. 16a). As expected, TCID treatment strongly inhibited collagen accumulation and the expression of the chemokines CCL2/7/12 (Fig. 5j, k, and Supplementary Fig. 16b). Furthermore, the mRNA levels of M2 macrophage markers and CD206-positive macrophages were decreased in the lungs of TCID-treated mice, whereas the expression of antifibrotic markers was increased (Supplementary Fig. 16c, d). We also observed that BLM-induced UCHL3 and p300 colocalization was significantly decreased by TCID treatment in ATII cells in mouse lungs (Fig. 5l, m). Furthermore, the colocalization of p300 and C/EBPβ decreased after the mice were injected with TCID (Supplementary Fig. 16e). Taken together, these results suggest that targeting p300 activity or stability may be an effective way to inhibit or treat pulmonary fibrosis.

## Discussion

IPF is a fatal interstitial lung disease for which no cure currently exists. Although two drugs have been approved for IPF treatment in several countries^35^, the survival of IPF patients remains poor. IPF is initiated by inflammation, followed by the massive production of fibrous connective tissue in the interalveolar septa^36^. This fibrotic process results in an excessive number of fibroblasts, an increase in lung collagen levels, the abnormal spatial distribution of ECM proteins, and ultimately, the deterioration of lung function^37^. To date, the relationship between the development of IPF and inflammation remains unclear. Although the initial inflammatory response is thought to initiate a fibrotic response in patients with IPF, this hypothesis remains controversial because immunosuppressive therapies are not effective in the treatment of IPF patients^4,14^. However, proinflammatory cytokines such as interleukin 1 and tumor necrosis factor-α and chemokines (CCL2/CCL7) are known to induce fibrosis in patients with IPF^38,39^. Thus, the interrelationship between the inflammatory process and fibrosis development in IPF remains unclear. Recently, increasing lines of evidence have indicated that ATII cells drive IPF and play a central role in pulmonary fibrosis^6,7^. Furthermore, ATII cells have been shown to secrete various inflammatory cytokines following repetitive lung injury, leading to fibroblast activation and ECM accumulation^40^. Therefore, understanding the role of ATII cells in the regulation of inflammation and fibrosis development is likely to lead to more significant advances in our understanding of IPF pathology. Our study demonstrated for the first time that p300 specifically activated ATII cell-derived chemotaxis signaling, causing M2 macrophage polarization and resulting in pulmonary fibrosis development. Furthermore, we suggested an alternative and promising target for IPF treatment by showing that ATII cell–mediated chemotaxis and fibrosis induction could be blocked by the selective inhibition of p300 activity or stability.

Fibrotic diseases are believed to be caused by the chronic accumulation of various genetic and environmental factors^1^. IPF and nonalcoholic fatty liver disease are thought to be associated with the abnormal expression of pro- or antifibrotic genes, which is mediated by epigenetic regulatory enzymes. To date, most studies have performed genetic profiling using fibrotic tissues; however, epigenetic approaches to disease progression are relatively incomplete. p300 is a key component of the epigenetic machinery that participates in the regulation of chromatin organization and transcription initiation^41,42^. The expression of p300 and its functional contributions to physiological responses are controlled by regulating cell type–specific expression and posttranslational modifications, and p300 may play important roles in fibrosis and regulation of the fibrotic response by controlling ECM homeostasis, myofibroblast activation, and the epithelial–mesenchymal transition^21^. Thus, we observed that the expression of HAT proteins other than p300 was not altered in the lung tissue of patients with IPF compared to normal controls, suggesting a plausible role of p300 as a main epigenetic regulator during the development of pulmonary fibrosis. The HAT activity of p300 and its interaction with activated Smads are essential for TGF-β_1_–induced profibrotic signaling, demonstrating that p300 might play a critical role in the progression of tissue fibrosis^29,43^. We recently identified a developmental mechanism for endometriosis fibrosis associated with epigenetic imbalance and suggested that p300 was a potential new target for endometriosis^44^. Although increasing evidence suggests that ATII cells play a pivotal role in IPF, no studies have been conducted examining the epigenetic regulatory mechanisms that are active in ATII cells or the mechanisms associated with lung fibrosis induction. In this study, we suggested a new molecular model for p300-mediated transcriptional regulation of chemokine genes in ATII cells. C/EBPβ was shown to be a p300-associated factor, leading to the transcriptional activation of chemokine genes, and the colocalization of p300 with C/EBPβ was significantly increased in the lungs of patients with IPF and lung fibrosis model mice. Moreover, we found that UCLH3 specifically bound to and stabilized p300, thereby activating p300–dependent transcriptional activation of chemokine genes via C/EBPβ. Selective inhibition of UCHL3 by TCID significantly reversed collagen deposition and the increase in M2 macrophage markers induced by BLM injection. Moreover, selective inhibition of UCHL3 impaired BLM-induced colocalization of p300 and C/EBPβ in ATII cells. These data indicate that UCHL3 mediates p300/C/EBPβ-dependent chemokine signaling in ATII cells. Although several previous studies have demonstrated the reversible ubiquitination of p300^45,46^, there have been no reports of E3 ligase-mediated p300 ubiquitination. Further studies are required to identify the E3 ligase that mediates the ubiquitination of p300 and examine the detailed mechanisms underlying the removal of p300 ubiquitination of UCHL3.

Pulmonary fibrotic diseases are often associated with the arrest of monocytes, neutrophils, mast cells, and other leukocytes^47^, and the release of chemokines by these proinflammatory cells and resident cells (alveolar epithelial cells) enhances inflammation and fibrosis in the lung. CCL2 is the most extensively studied chemokine associated with lung fibrosis^48^. An increase in CCL2 has been identified in BAL fluid and serum samples derived from patients with IPF^49,50^. Moreover, alveolar epithelial cells within fibrotic areas have been reported to exhibit increased CCL2 expression in patients with IPF^48^. Despite the important role of chemokines in IPF pathogenesis, studies of CCL2-deficient mice and clinical trials of a monoclonal antibody that blocks CCL2 have failed^51^. In this study, it was observed that the amount of total CCL2 in the serum of subjects who received CCL2 monoclonal antibodies was significantly increased compared to that in the placebo-treated group, suggesting that there was a compensatory mechanism. CCL7 is expressed at significantly increased levels in biopsied tissues from patients with IPF compared with normal samples^39^. CCL12, the CCL2 analog expressed in humans, was also elevated in the lungs of a fibrosis mouse model^38^. Compensatory increases in CCL2 and CCL7 expression were also observed in *Ccl12*-knockout mice. These results indicate that chemokines affect the progression of lung fibrosis by activating compensatory actions among each other. Intriguingly, lung fibrosis was efficiently inhibited in ATII cell-specific *Ccl12* knockout mice, and the expression levels of CCL2 and CCL7 were decreased in BAL fluid obtained from these mice^52^. Therefore, the regulation of chemokine signals in ATII cells appears to be critical for the treatment of lung fibrosis. Previous studies have shown that blocking the signaling of a single chemokine is inefficient due to the presence of compensatory actions. Here, we suggest that p300 can serve as a master chemokine regulator in ATII cells. In addition, we demonstrated that blocking p300 activity or stability in ATII cells prevented compensatory actions among C-C chemokines, leading to the suppression of pulmonary fibrosis. Recent studies have shown that p300 is a promising target for the treatment of fibrotic diseases such as lung fibrosis and liver fibrosis^26,53^. Knowledge of these mechanisms will be necessary for the development of strategies to treat IPF, and further preclinical research will be required to investigate whether inhibiting p300 activity or stability may be effective for this purpose.

In summary, we demonstrated that p300 in ATII cells mediated chemokine signaling to induce the infiltration of activated M2 macrophages, leading to lung fibrosis (Supplementary Fig. 17). In particular, we found that UCHL3-mediated deubiquitination of p300 led to the transcriptional activation of the chemokines *Ccl2, Ccl7*, and *Ccl12* through the cooperative action of p300 and C/EBPβ, which consequently promoted M2 macrophage polarization in an ATII cell-specific manner. Finally, we provided a basis for the future development of a novel IPF therapy based on the inhibition of p300 activity or stability. Collectively, our study offers a conceptual framework for understanding the role of p300 in ATII cells, which has implications for the diagnosis and treatment of IPF.

## Supplementary information

Supplementary information

## Data Availability

The RNA-sequencing and ChIP-sequencing data have been deposited in the NCBI Gene Expression Omnibus and are accessible through the GEO series using accession numbers GSE190157 and GSE190150, respectively. All other data supporting the findings of this study are available upon reasonable request. Source data are provided with this paper.
