# Supplementary material for: The deubiquitinase UCHL3 mediates p300-dependent chemokine signaling in alveolar type II cells to promote pulmonary fibrosis
Source: Exp Mol Med. 2023 Aug 1;55(8):1795–805. doi: 10.1038/s12276-023-01066-1 (PMC10474292; doi:10.1038/s12276-023-01066-1)
Supplement: Supplementary file 1 — Supplementary information [file 12276_2023_1066_MOESM1_ESM.pdf]

Supplementary informations for

The deubiquitinase UCHL3 mediates p300-dependent chemokine signaling in alveolar type II cells to promote pulmonary fibrosis

Soo Yeon Lee[1,†], Soo-Yeon Park[1,†], Seung-Hyun Lee[1], Hyunsik Kim[1], Jae-Hwan Kwon[1], Jung-Yoon Yoo[2], Kyunggon Kim[3], Moo Suk Park[4], Chun Geun Lee[5,6], Jack A. Elias[5], Myung Hyun Sohn[7], Hyo Sup Shim[8,*], and Ho-Geun Yoon[1,*]

*Correspondence to: YHGEUN@yuhs.ac, shimhs@yuhs.ac

This PDF file include:

SUPPLEMENTARY MATERIALS AND METHODS

Supplementary Table 1 to 3

Supplementary Figure 1 to 17

SUPPLEMENTARY MATERIALS AND METHODS

RNA-sequencing

Total RNA was isolated using TRIzol reagent (Invitrogen, Waltham, MA, USA). RNA quality was assessed using an Agilent 2100 bioanalyzer using the RNA 6000 Nano Chip (Agilent Technologies, Amstelveen, Netherlands), and RNA quantification was performed using ND-2000 Spectrophotometer (Thermo Fisher Scientific, Waltham, MA, USA).

For control and test RNAs, library construction was performed using QuantSeq 3'mRNA-Seq Library Prep Kit (Lexogen BioCenter, Vienna, Austria), according to the manufacturer's instructions. In brief, 500 ng total RNA was prepared for each sample, an oligo-dT primer containing an Illumina-compatible sequence at its 5'end was hybridized to the RNA, and reverse transcription was performed. After degradation of the RNA template, second-strand synthesis was initiated by a random primer containing an Illumina-compatible linker sequence at its 5'end. The double-stranded library was purified by using magnetic beads to remove all reaction components. The library was amplified to add the complete adapter sequences required for cluster generation. The finished library is purified from PCR components. High-throughput sequencing was performed as single-end 75 sequencing using NextSeq 500 (Illumina, San Diego, CA, USA).

ChIP-sequencing

Whole lungs of bleomycin- and PBS-treated mice were used for ChIP-seq analyses, which were performed by Active Motif (Carlsbad, CA, USA). The samples were fixed and subjected to immunoprecipitation using an antibody against p300 (Santa Cruz Biotechnology, Dallas, TX, USA). In brief, the 75-nt single-end (SE75) sequence reads generated by Illumina sequencing (using NextSeq 500) were mapped to the genome using the Burrows–Wheeler Aligner algorithm with default settings. Reads were extended at their 3'-ends to a length of

200 base pair fragments in a size-selected library. The densities of fragments (signal map) were determined by the number of fragments in each 32-nt bin along the genome. The tag number of samples within a comparison group was normalized by random sampling against the number of tags in the smallest sample. Data were visualized by the University of California, Santa Cruz (UCSC) genome browser. EnrichR was used for pathway analysis.

Harvesting and fixation of lungs

An incision was made in the abdomen, and the rib cage was cut open to expose the heart and lungs, and the lungs were perfused with 10 mL PBS through the pulmonary artery. The left lobe of the mouse lung from each group was fixed in formaldehyde (4% w/v) for 48 hours at room temperature, followed by paraffin embedding. Paraffin blocks were sectioned by the Histology Core at the Yonsei University of Medicine, Seoul, South Korea.

Masson's trichrome staining

To confirm collagen production, sections were deparaffinized, hydrated with water, and stained in Bouin's solution (Merck, Darmstadt, Germany) for 1 minute using the microwave and then allowed to stand for 15 minutes at room temperature. After washing in running tap water, the sections were placed in hematoxylin for 10 minutes, rinsed in running tap water, and stained in Biebrich scarlet for 5 minutes. The sections were then stained in phosphotungstic/phosphomolybdic acid for 15 minutes, transferred directly into aniline blue for 5 minutes, dehydrated, and coverslipped. Blue-stained collagen fiber quantification was performed using ImageJ software in a ×200 field image area (Olympus, Tokyo, Japan).

Immunostaining

In preparation for IHC, mouse lung section slides were rehydrated through a graded alcohol series, followed by antigen retrieval in 10 mM sodium citrate. The IHC staining protocol included the sequential application of a peroxidase-blocking reagent, primary antibody, secondary antibody molecules and horseradish peroxidase coupled to a polymer

backbone, and visualization using a 3,3’-diaminobenzidine tetrahydrochloride (DAB) chromogen reagent with a hydrogen peroxide substrate (Dako, Santa Clara, CA, USA). The following primary antibodies were used: p300 (Abcam, Cambridge, MA, USA), PCAF (Abcam, Cambridge, MA, USA), CBP (Cell signaling, Danvers, MA, USA), TIP60 (Santa Cruz Biotechnology, Dallas, TX, USA), and GCN5 (Santa Cruz Biotechnology, Dallas, TX, USA).

For immunocytochemistry, the sample preparation process prior to antibody application was the same process used for IHC. The following primary antibodies were used: p300 (Santa Cruz Biotechnology, Dallas, TX, USA), SP-C (Santa Cruz Biotechnology, Dallas, TX, USA), CCSP (Merck, Darmstadt, Germany), PDPN (R&D, Minneapolis, MN, USA), Muc5AC and FOXJ1 (Thermo Fisher Scientific, Waltham, MA, USA), α-SMA (Abcam, Cambridge, MA, USA), and C/EBPα and C/EBPβ (Santa Cruz Biotechnology, Dallas, TX, USA), UCHL3 (Abcam, Cambridge, MA, USA). The following secondary antibodies were used: DyLight 488 anti-rabbit (Vectorlab, Burlingame, CA, USA) and DyLight 549 anti-mouse (Vectorlab, Burlingame, CA, USA). All secondary antibodies were Alexa Fluor-coupled. After staining, slides were mounted with a mounting medium containing 4’,6-diamidino-2-phenylindole (DAPI; Abcam, Cambridge, MA, USA) and stored at 4°C in the dark. Slides were imaged and analyzed using a Zeiss LSM780 confocal microscope (Carl Zeiss, Oberkochen, Germany).

Chromatin immunoprecipitation assay

Chromatin immunoprecipitation (ChIP) assay was carried out on lung samples and RLE-6TN cells using a Pierce Agarose ChIP kit (Thermo Fisher Scientific, Waltham, MA, USA) according to the manufacturer's instructions. The nuclear lysates were precipitated with p300 or C/EBPβ antibody (Santa Cruz Biotechnology, Dallas, TX, USA) or normal mouse IgG (Thermo Fisher Scientific, Waltham, MA, USA) overnight at 4C together with ChIP

grade protein A/G agarose beads. The beads were washed, eluted, and treated with proteinase K. The recovered DNA was amplified by qPCR. The primers used in ChIP assay was indicated in Supplementary Table 3.

***In situ* proximity ligation assay analysis**

In situ PLA analysis (Merck, Kenilworth, NJ, USA) was performed according to the manufacturer's instructions. Cells were fixed with 4% paraformaldehyde, washed with PBS, and blocked with blocking solution. For paraffin-embedded tissue samples, slides were rehydrated in xylene and ethanol, and citrate buffer was used for antigen retrieval. After the application of acetyl-lysine and C/EBPβ antibodies, samples were incubated with PLUS and MINUS secondary PLA probes, based on the antibody species, and subjected to ligation and amplification using the provided reagents. The samples were mounted with Duolink mounting medium and analyzed using an LSM 780 Laser Scanning Microscope (Carl Zeiss, Oberkochen, Germany).

Flow cytometry analysis and sorting

For RNA-seq analysis, primary murine ATII cells were obtained from 8–10-week-old C57BL/6 and ATII cell–specific *p300* KO mice. Mice were sacrificed, and the lungs were perfused and cannulated, as described above. To seal the upper airway, 2 mL dispase (Stemcell, Vancouver, Canada) was instilled into the lungs. The lungs were removed and incubated in dispase at room temperature for 40 minutes. After enzymatic digestion with dispase, lung samples were gently minced with a GentleMACS Dissociator (Miltenyi Biotec, Bergisch Gladbach, Germany). Cells were first incubated with red blood cell lysis buffer. After spinning down, cells were blocked in. Cells were then stained with different antibodies. Antibodies used: Ep-CAM-APC (Invitrogen, Waltham, MA, USA), CD74-FITC (BD Biosciences, San Jose, CA, USA). Sorting was performed on a BD FACS Aria^{TM} III flow cytometer (BD Biosciences, San Jose, CA, USA). One mouse was used for each

isolation, and the typical yield was approximately 1.0×10^6 primary ATII cells per animal. Cell purity was determined via immunocytochemical staining of samples using surfactant protein C (SPC) to label primary ATII cells.

To examine M2 macrophage polarization, flow cytometry analysis was conducted. Single lung cells were prepared, as described above. After blocking, cells were stained with antibodies for CD45-AF488 (BioLegend, San Diego, CA, USA), F4/80-BV605 (BD Biosciences, San Jose, CA, USA), and CD206-APC780 (Invitrogen, Waltham, MA, USA).

Soluble collagen assay

Collagen contents in the mouse lung samples were determined biochemically using the Sircol Collagen Assay Kit (Biocolor, Carrickfergus, United Kingdom). In brief, 100 mg of lung samples were homogenized, the supernatant was collected, and collagen binding dye was added and incubated for 1 hour at room temperature, followed by 10,000 x *g* centrifugation. The visible red pellet was dissolved in 100% ethanol to remove excess dye and subjected to additional centrifugation. Subsequently, the pellet was dissolved in 0.5 M sodium chloride solution, incubated for 30 minutes at 37°C, and absorbance was measured at 540 nm using a spectrophotometer.

RNA isolation and quantitative reverse transcription–polymerase chain reaction

Total RNA was extracted using TRIzol reagent, following the standard protocol (Takara, Kyoto, Japan), and cDNA was synthesized using CellScript (CellSafe, Seoul, Korea), following the manufacturer’s protocol. The concentration of cDNA was normalized using *GAPDH*. qRT-PCR analyses were performed using SYBR Green PCR master mix reagents and an ABI Prism 7700 sequence detection system (Applied Biosystems, Carlsbad, CA, USA). All reactions were performed in triplicate. Relative expression levels and standard deviations of target genes were calculated using the comparative method. The primers used in qRT-PCR was indicated in Supplementary Table 2.

Quantification of cytokines

Cytokine levels in mouse serum and BAL fluid were assayed by enzyme-linked immunosorbent assays (ELISAs). ELISAs were performed for the following mouse protein: CCL2 (R&D Systems), CCL7 (MyBioSource), and CCL12 (R&D Systems).

Cell culture and reagents

Rat lung alveolar type 2 cell line RLE-6TN, mouse lung club cell line C22, mouse fibroblast cell line MLg, and mouse lung alveolar macrophage cell line MH-S were purchased from Korean Cell Line Bank (Seoul, Korea). RLE-6TN and MH-S were cultured in Dulbecco's modified Eagle's medium (DMEM) supplemented with 10% (v/v) fetal bovine serum (FBS) and 1% antibiotic/antimycotic solution (Corning, Manassas, VA, USA) at 37°C under 5% CO_2. MH-S were grown in complete DMEM supplemented with 50 μM 2-mercaptoethanol. C22 were maintained in permissive conditions [DMEM supplemented with 4% FBS, penicillin (100 U/ml), streptomycin (100 μg/ml), 2 mM glutamine, endothelin-1 (0.25 μg/ml), interferon-γ (0.01 μg/ml), insulin (10 μg/ml), transferrin (5 μg/ml), endothelial cell growth supplement (7.5 μg/ml), epidermal growth factor (0.025 μg/ml), hydrocortisone (0.36 μg/ml), and T_3 (0.02 μg/ml)] at 33°C under 5% CO_2. Bleomycin was purchased from Santa Cruz Biotechnology (Dallas, TX, USA). C646 was purchased from Sigma-Aldrich (St. Louis, MO, USA). TGF-β_1 was purchased from ProSpec (East Brunswick, NJ, USA).

Construction of stably cell lines

To generate the cell lines for the stable knockdown of *p300*, lentiviral vectors (scramble, shp300) used in this study were purchased from Sigma-Aldrich, U.S.A. The auxiliary plasmid liposomes (pxPAX.2 and Pmd2.G) and lentivirus vector were transfected into 293FT cells to produce lentivirus by using Lipofectamine 3000 transfection reagent (Invitrogen, Carlsbad, CA, USA). After 48 hours of transfection, the supernatant was collected and centrifuged to discard cell debris. The centrifuged supernatant was filtered by

using a 0.45 μm polyvinylidene difluoride filter. RLE-6TN cells were infected with viral suspension mixed with 8 μg/ml polybrene. After 48 hours of infection, 2 μg/ml puromycin was added to screen stably transduced cell lines.

Plasmids, Site-directed mutagenesis and small interfering RNAs (siRNAs)

Rat C/EBPβ LAP (Liver-enriched activator protein) form-pcDNA3.1 construct was obtained from Addgene (Watertown, MA, USA). Site-directed mutagenesis was performed to make K39R mutant using with 18 PCR amplification cycles under the following reaction conditions: denaturation at 94°C for 30 s, annealing at 55 °C for 1 min, and extension at 68°C for 10 min. Amplified mixtures were treated with *Dpn*I (Agilent Technologies) at 37°C for 1 h, and aliquots were used to transform competent *E. coli*. All constructs were confirmed by DNA sequencing. For siRNA transfection, cells were plated at 60-70% confluency and transfected using Lipofectamine RNAiMax (Life Technologies, Carlsbad, CA, USA) with 10 pM siRNA following the manufacturer's protocol. The medium was changed after 4 h, cells were incubated for 2 days, and then treated with the indicated reagents. The following siRNA sequences were used: for siCebpa #1, sense 5'-CCAAGAAGUCGGUGGAUAAUU -3' and antisense 5'-UUAUCCACCGACUUCUUGGUU-3'; for siCebpa #3, sense 5'-GGAGUUGACCAGUGACAAUUU-3' and antisense 5'-AUUGUCACUGGUCAACUCCUU-3'; for siCebpb #3, sense 5'-GGUUUCGGGACUUGAUGCAUU -3' and antisense 5'-UGCAUCAAGUCCCGAAACCUU-3'; siCebpb #4, sense 5'-GCAACACACGUGUAACUGUUU -3' and antisense 5'-ACAGUUACACGUGUGUUGCUU-3'; for negative control, sense 5'-CCUCGUGCCGUUCCAUCAGGUAGUU-3' and antisense 5'-CUACCUGAUGGAACGGCACGAGGUU -3'.

Luciferase reporter gene assay

To make *Ccl2*/*Ccl7*/*Ccl12* gene promoter constructs, p300-BE regions of each gene were prepared by PCR amplification of mouse genomic DNA obtained from C22 cells. The amplified product was purified and cloned into pGL3.0. This chemokine's reporter gene was used as a template for all subsequent mutation constructs. To perform the luciferase reporter assay, the cells were transiently transfected with 0.5 ug of chemokine reporter gene in 24-well plates. After 24 hours of incubation, the cells were treated with 10 ng/mL of TGF-β_1 in serum-free medium for another 24 hours. The cells were harvested and lysed to assay luciferase activity using a luciferase assay system (Promega, Madison, WI, USA). Luminescence was measured by a MicroLumatPlus LB96V Microplate Luminometer (Berthold, Bad Wildbad, Germany).

Western blot analysis and immunoprecipitation.

Mouse lung and RLE-6TN cells were lysed in lysis buffer (20mM Tris-Cl, 150mM NaCl, 1% Triton X-100, 1.5% $MgCl_2$, 1mM ethylenediaminetetraacetate, 1 mM Na_2VO_4, 1mM phenylmethylsulfonyl fluoride, and protease inhibitor cocktail at pH 7.5). Lysates were briefly vortexed and cleared by centrifugation at 16,000 × g for 20 min at 4°C; supernatants were collected and transferred to fresh tubes. Protein concentrations were determined using a 660-nm protein assay reagent (Thermo Fisher Scientific, Waltham, MA, USA). For immunoprecipitation assay, the lysates were then precleared with protein A/G agarose beads (Santa Cruz Biotechnology, Dallas, TX, USA) for 2 h and precipitated using protein A/G agarose beads. Equal amounts of protein extracts and immunoprecipitation products were subjected to electrophoresis on sodium dodecyl sulfate-polyacrylamide gels and transferred to nitrocellulose transfer membranes (Whatman, Dassel, Germany). The membranes were blocked in Tris buffer (pH 7.4) containing 0.1% (v/v) Tween 20 (Sigma-Aldrich) and 5% (w/v) nonfat Difco skim milk (BD Biosciences, San Jose, CA, USA), and then probed with primary antibodies. The following antibodies were used: anti-p300, anti C/EBPβ, UCHL3 and anti-

C/EBPα (Santa Cruz Biotechnology, Dallas, TX, USA); Anti-HA, p-Smad3 and Lamin A/C antibodies (Cell Signaling, Danvers, MA, USA); anti-β-actin (Sigma-Aldrich, St. Louis, MO, USA). The membranes were then washed with 1× PBST, incubated with the appropriate secondary anti-rabbit or anti-mouse horseradish peroxidase-conjugated antibodies (Thermo Fisher Scientific, Waltham, MA, USA) for 1 h, and visualized using the LAS-3000 system (Fujifilm, Stamford, CT, USA) with an enhanced chemiluminescence detection reagent (Thermo Fisher Scientific, Waltham, MA, USA).

Supplementary Table 1: Demographic and clinical data of study population.

	Control (n=16)	IPF (n=42)	*P*-value
Age, years	62.0 (34.0-83.0)	60.5 (31.0-69.0)	0.1314
Height, cm	160.5 ± 9.6	167.0 ±7.7	0.1091
Weight, kg	66.5 ± 16.7	62.0 ± 11.1	0.2997
IPF stage, I:II:III (n)	N/A	1:20:21	
FVC, L	3.0 ± 0.6	1.4 ± 0.5	<0.0001
FVC, % predicted	94.5 ± 12.8	37.0 ± 11.1	<0.0001
FEV_1, L	2.2 ± 0.5	1.3 ± 0.6	<0.0001
FEV_1, % predicted	102.5 ± 14.3	47.0 ± 21.9	<0.0001
DLCO, mL/mmHg/min	18.1 ± 3.1	5.4 ± 6.2	<0.0001
DLCO, % predicted	95.5 ± 13.2	28 ± 10.6	<0.0001
p300 intensity/H&E	0.7293 (0.0844-2.4565)	2.536 (0.6019-12.2473)	<0.0001

FVC, Forced Vital Capacity; FEV_1, Forced Expiratory Volume in 1 s; DLCO, Diffusing Capacity for Carbon Monoxide. N/A, not applicable. Data are given as number (%), mean (± standard deviation), or median (interquartile range), as appropriate.

Supplementary Table 2: List of primers used in qRT-PCR analysis.

Gene		Sequence
rat *Gapdh*	Forward	5’-TGATTCTACCCACGGCAAGTT-3’
	Reverse	5’-TGATGGGTTTCCCATTGATGA-3’
rat *Ccl2*	Forward	5’-CAGATGCAGTTAACGCCCCA-3’
	Reverse	5’-TTGAGCTTGGTGACAAAAACTACAG-3’
rat *Ccl7*	Forward	5’-GCACCGAGTCTGCCAACTTT-3’
	Reverse	5’-GGATGAATTGGTCCCATCTGGT-3’;
rat p300	Forward	5’-CCAGCCCAGACCAGAATTCAAT-3’
	Reverse	5’-CTAGTGTACTCTGTGAGAGGTTTGAA-3’
rat Cebpa	Forward	5’-GTCACTGGTCAACTCCAGCAC-3’
	Reverse	5’-CAAGAACAGCAACGAGTACCG-3’
rat Cebpb	Forward	5’-GGAGACGCAGCACAAGGT-3’
	Reverse	5’-AGCTGCTTGAACAAGTTCCG-3’
mouse *Gapdh*	Forward	5’-CGACTTCAACAGCAACTCCCACTCTTCC-3’
	Reverse	5’-TGGGTGGTCCAGGGTTTCTTACTCCTT-3’
mouse p300	Forward	5’-CTGTGAACAACATGAGTGCTAGTCC-3’
	Reverse	5’-TGAGCTGCTGTTGGCAAAGG-3’
mouse *Ccl2*	Forward	5’-CAGATGCAGTTAACGCCCCA-3’
	Reverse	5’-TTGAGCTTGGTGACAAAAACTACAG-3’
mouse *Ccl7*	Forward	5’-GAGTCTGCCAGCTCTCACTG-3’
	Reverse	5’-GCATTGGGCCCATCTGGTTG-3’
mouse *Ccl12*	Forward	5’-CCGGGAAGCTGTGATCTTCAGG-3’
	Reverse	5’-TGGGGAACTTCAGGGGGAAAT-3’
mouse *Arg1*	Forward	5’-AAGAATGGAAGAGTCAGTGTGG-3’
	Reverse	5’-GGGAGTGTTGATGTCAGTGTG-3’
mouse *Cd206*	Forward	5’-ATGGATGTTGATGGCTACTGG-3’
	Reverse	5’-TTCTGACTCTGGACACTTGC-3’
mouse *Cd163*	Forward	5’-AGTCATCTGCACTGGGAAAG-3’
	Reverse	5’-CAGTTTTCTTTGTGGGCTTCG-3’
mouse *Cox-2*	Forward	5’-CTGGGGGAAGAAATGTGCCA-3’
	Reverse	5’- GCCATTTCCTTCTCTCCTGTAAGT-3’
mouse *Cxcl10*	Forward	5’-CCACGTGTTGAGATCATTGCC-3’
	Reverse	5’- GAGGCTCTCTGCTGTCCATC-3’

Supplementary Table 3: List of primers used in ChIP assay

Gene		Sequence
mouse *Ccl2* #1	Forward	5'-CCTTGCTGAGTATCTCCTAAATGC -3'
	Reverse	5'- CGTTTCAAGCCATACTTTCTTCCAA -3'
mouse *Ccl2* #2	Forward	5'- TGGAGGATCACAGTAGCTGTCA -3'
	Reverse	5'- GGACAAGGATGGAGAAGAGAGTC -3'
mouse *Ccl2* #3	Forward	5'- CGGGAAACCCCAGCACGA -3'
	Reverse	5'- CTCTTAAACGGCAGTGTTTGATTCA -3'
mouse *Ccl2* #4	Forward	5'- CACTGTTCTAAGAGACTGGCAG -3'
	Reverse	5'- AGAGTAGTCTAAATCCTGACCCCTT -3'
mouse *Ccl2* #5	Forward	5'- TGCAAAAGAGAGAAAACAGACCCT -3'
	Reverse	5'- TTTAGCATCTGGCCTCTCCGG -3'
mouse *Ccl2* #6	Forward	5'- TACTATTGCTCTGGGGAAAAAAAATCTG -3'
	Reverse	5'- ACGGCTCCGGTTCGCTTT -3'
mouse *Ccl2* #7	Forward	5'- GCATTTCACCCTTTGACAGCTAC -3'
	Reverse	5'- ATAATTTCTGGTTGAGTTTGACCATGGA -3'
mouse *Ccl7* #1	Forward	5'- CCCCCCCCCTACTCCCT -3'
	Reverse	5'- CTGGGCTGGCCTTCCTCC -3'
mouse *Ccl7* #2	Forward	5'- CCCTACATTCCAGGTCCAGT -3'
	Reverse	5'- CTTCGAGAAAAAGAAACCTAGTCAG -3'
mouse *Ccl7* #3	Forward	5'- TGGCTGATATTCCACTCTATACTTTTAC -3'
	Reverse	5'- TTAGTGCAGTCATTGTCAGGAA -3'
mouse *Ccl12* #1	Forward	5'- GTATCAGCAAAATGAAAAGGCAATC -3'
	Reverse	5'- TAATGTTTATTGGTGATGAGCTTATTT -3'
mouse *Ccl12* #2	Forward	5'- GGTTAAGAATATAATCACATAGTATACA -3'
	Reverse	5'- TCTCACCACTCACACTCCCT -3'
mouse *Ccl12* #3	Forward	5'- CACCCTCCAAAAGGCAGATTC -3'
	Reverse	5'- GCTGTACAGTTTCTGTCTACTG -3'

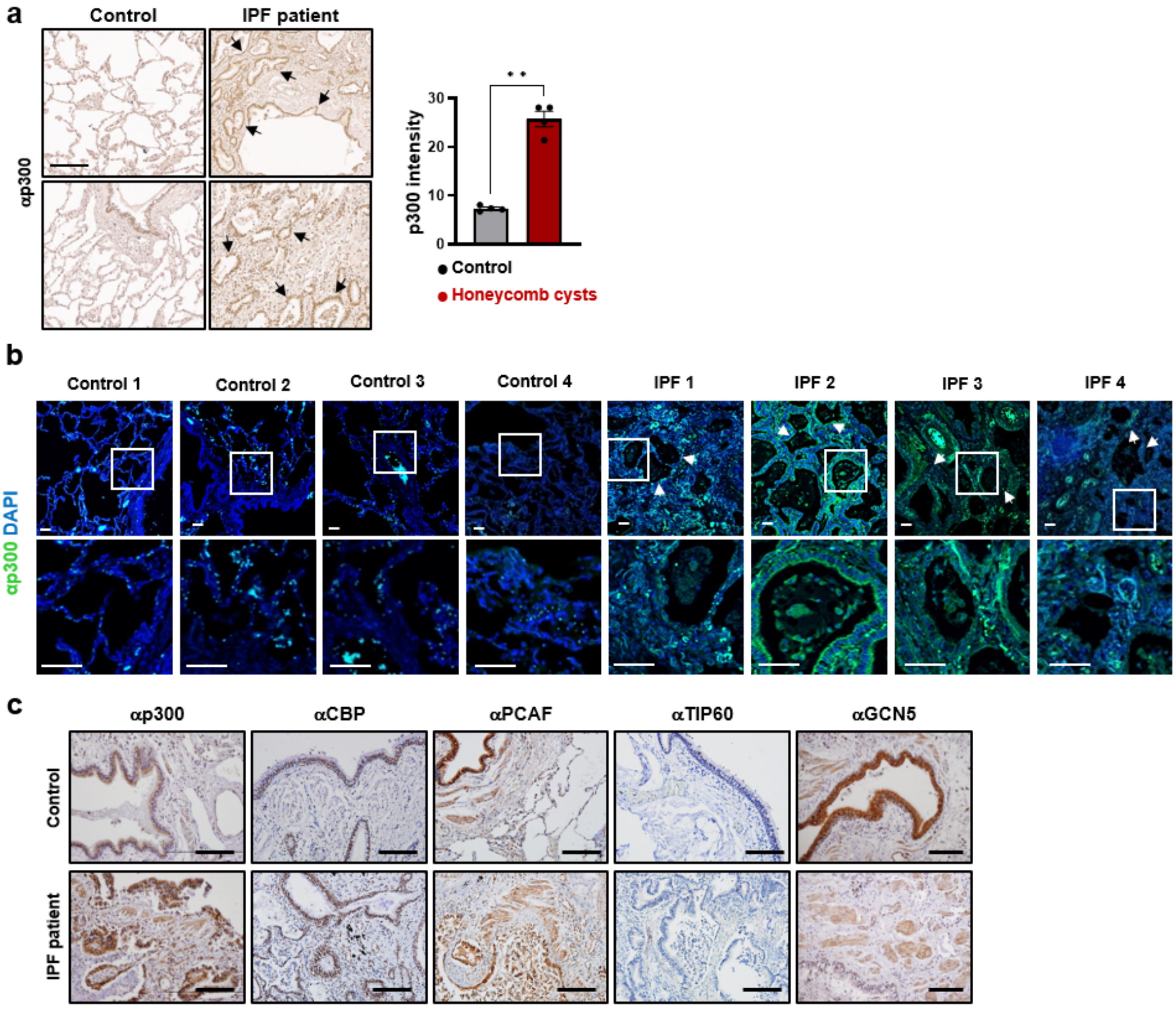

Supplementary Fig. 1: Increased p300 expression in the honeycomb region of lungs from patients with IPF.

a IHC staining for p300 protein in normal lung and honeycomb region of IPF lung. Black arrows indicate honeycomb cysts. p300 intensity was quantified using ImageJ software. Scale bars = 500 μm. n = 4 per each group. **P < 0.01, two-tailed t-test with Welch's correction. Error bars, mean ± s.e.m. **b** Immunofluorescence staining against p300 was performed in IPF lungs. White arrows indicate honeycomb cysts. Scale bars = 50 μm. **c** IHC staining for p300, CBP, PCAF, TIP60, GCN5 protein in lung samples from IPF patients and control subjects. Scale bar = 100 μm.

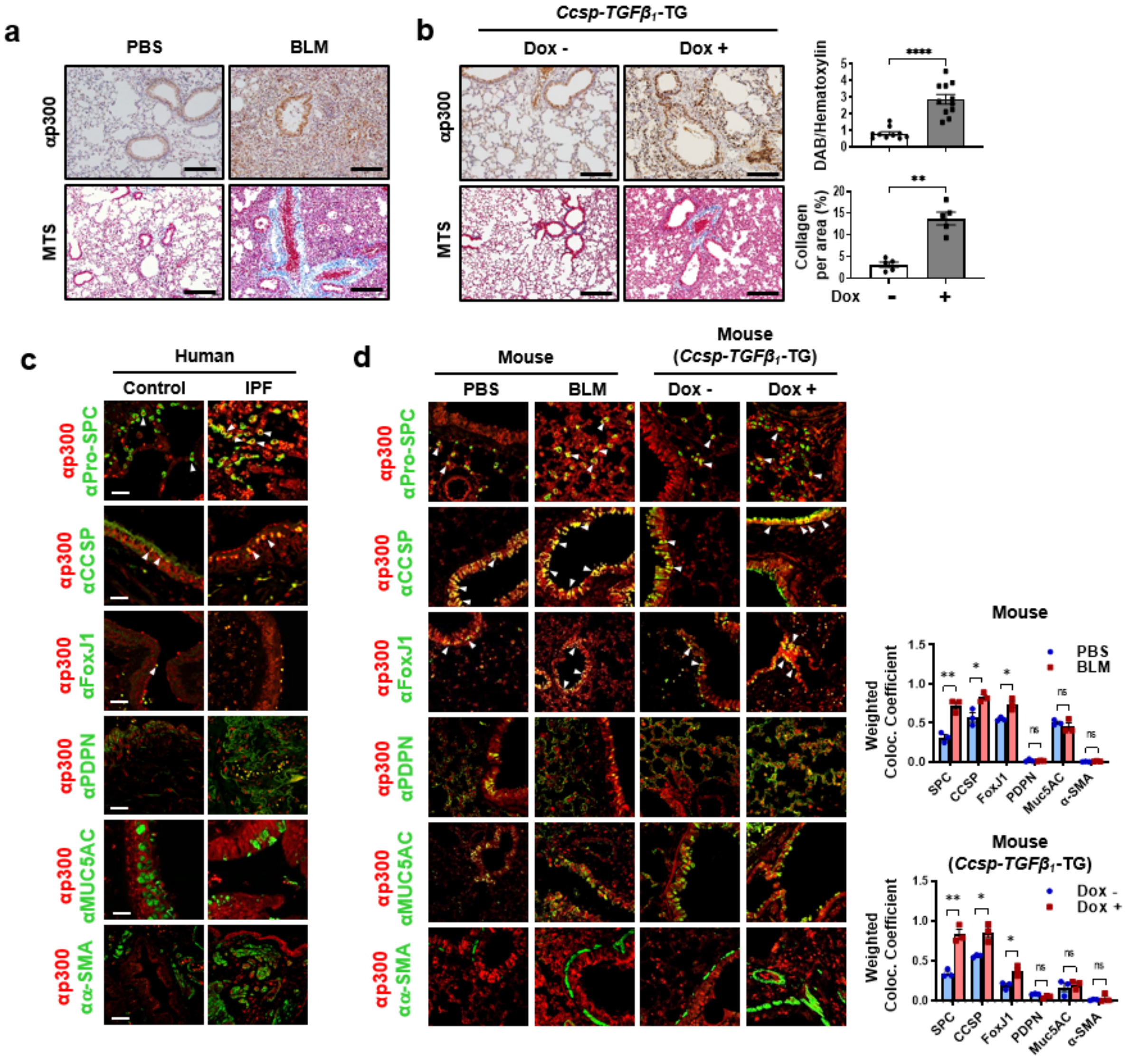

Supplementary Fig. 2: p300 expression is significantly increased in the lung epithelial cells of patients with IPF and lung fibrosis mouse models

a, b p300 IHC and MTS for collagen in lung samples from BLM-induced lung fibrosis (a) and *Ccsp-TGF-*β_1-TG model mice (b). Scale bar, 100 µm. Dot plot represents p300 intensity/Hematoxylin ratio or p300/blue region of MTS. n = 5–11 mice/group, two-tailed Mann–Whitney U test. $^{**}P < 0.01$; $^{****}P < 0.0001$. **c, d** Immunofluorescence assay in human (c) and mouse lungs (d) using the indicated antibodies. Arrowheads indicate co-localization of p300 and each specific cell marker. Scale bars, 50 µm. Cell markers are as follows: Pro-SPC, ATII cells; CCSP, club cells; FoxJ1, ciliated cells; PDPN, ATI cells; Muc5AC, goblet cells;

and α-SMA, fibroblasts. Weighted co-localized coefficient was calculated using ZEN 3.0 software. $n = 3$ per each group. All average data are mean ± s.e.m. n.s., not significant; $*P < 0.05$; $**P < 0.01$, two-tailed Mann–Whitney U test.

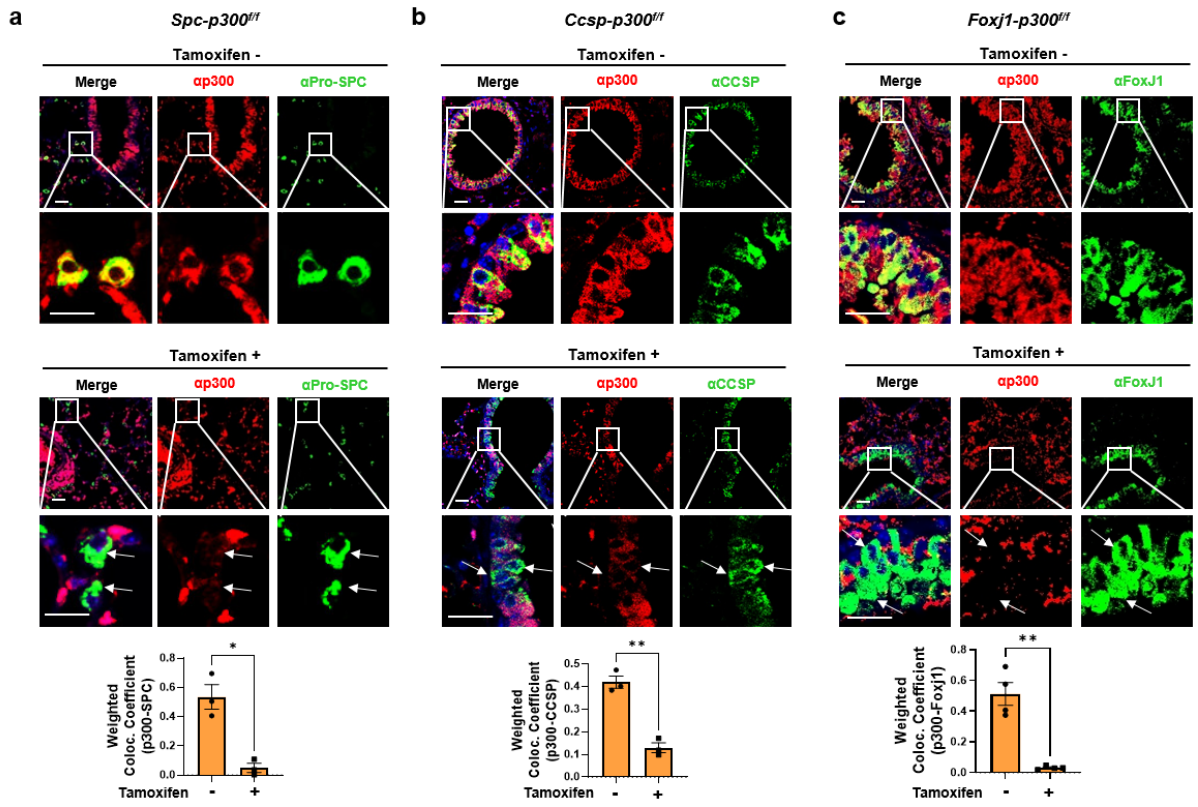

Supplementary Fig. 3: Validation of p300 ablation in the lungs of *Spc-p300$^{f/f}$*, *Ccsp-p300 $^{f/f}$*, and *Foxj1-p300 $^{f/f}$* mice.

Co-immunofluorescence analysis of p300 and cell-specific markers was performed in the lungs of (a) *Spc-p300$^{f/f}$*, (b) *Ccsp-p300 $^{f/f}$*, and (c) *Foxj1-p300 $^{f/f}$* mice to verify the deletion of p300. After tamoxifen was injected, colocalized areas were diminished (White arrows). Scale bars, 20 µm. Weighted co-localized coefficient was calculated using ZEN 3.0 software. Error bars, mean ± s.e.m. n = 3 per each group. n.s., not significant; *P < 0.05, **P < 0.01, two-tailed t test with Welch's correction.

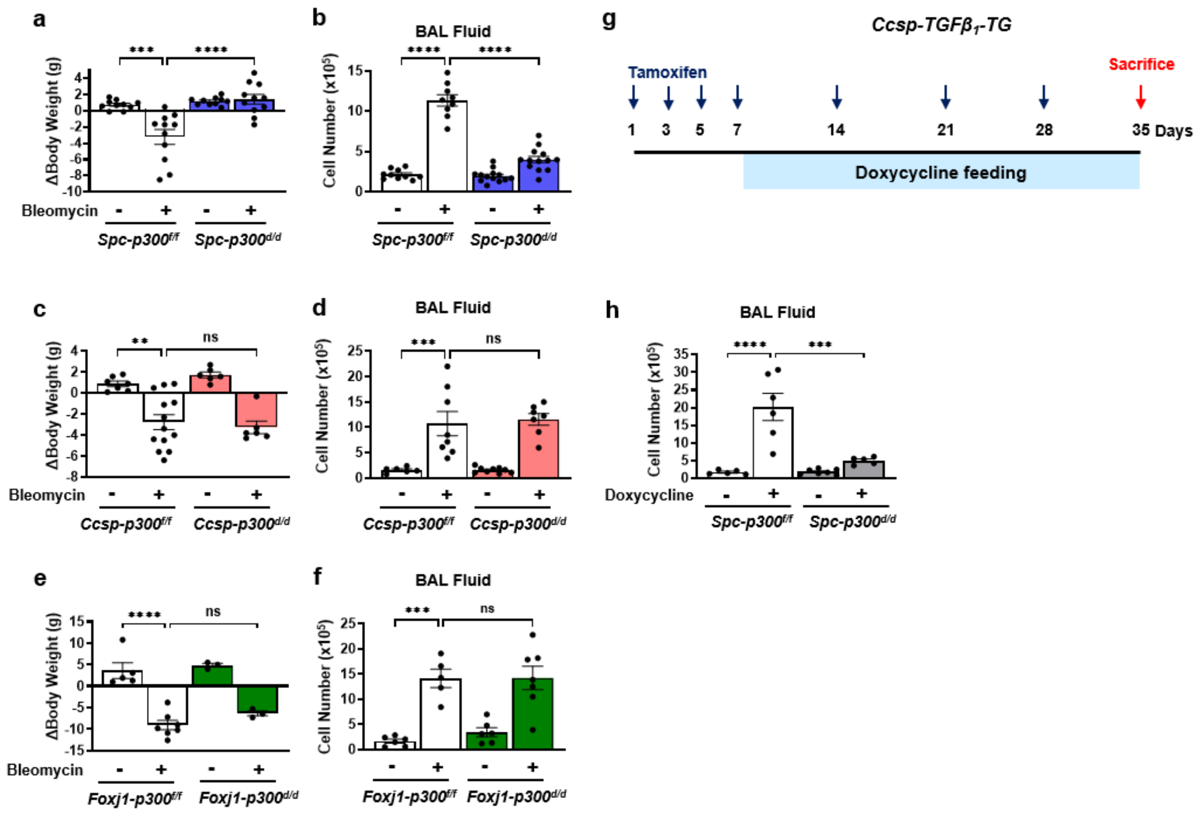

Supplementary Fig. 4: Effect of bleomycin treatment on body weight and BAL fluid cells count in control and epithelial cell–specific p300 knockout mice.

a, c, and e Body weight loss in PBS or BLM-treated (a) Spc-p300$^{f/f}$, (c) Ccsp-p300 $^{f/f}$, and (e) Foxj1-p300 $^{f/f}$ mice with or without tamoxifen. **b, d, f, and h** Total cell counts in BAL fluid were determined after the instillation of BLM or PBS in (b) Spc-p300$^{f/f}$, (d) Ccsp-p300 $^{f/f}$, (f) Foxj1-p300 $^{f/f}$, and (h) Spc-p300$^{f/f}$ in Ccsp-TGF-β_1-TG mice with or without tamoxifen. **g** Schematic of the experimental design and timeframe. 8–10-week-old control and Spc-p300$^{f/f}$ in Ccsp-TGF-β_1-TG mice were injected with tamoxifen 3 times for the first week, maintained on doxycycline throughout the course of the experiment. Error bars represent the mean ± s.e.m. of n = 3-15 mice per group. One-way ANOVA with Tukey's test. ns, not significant, **P < 0.01, ***P < 0.001 and ****P < 0.0001.

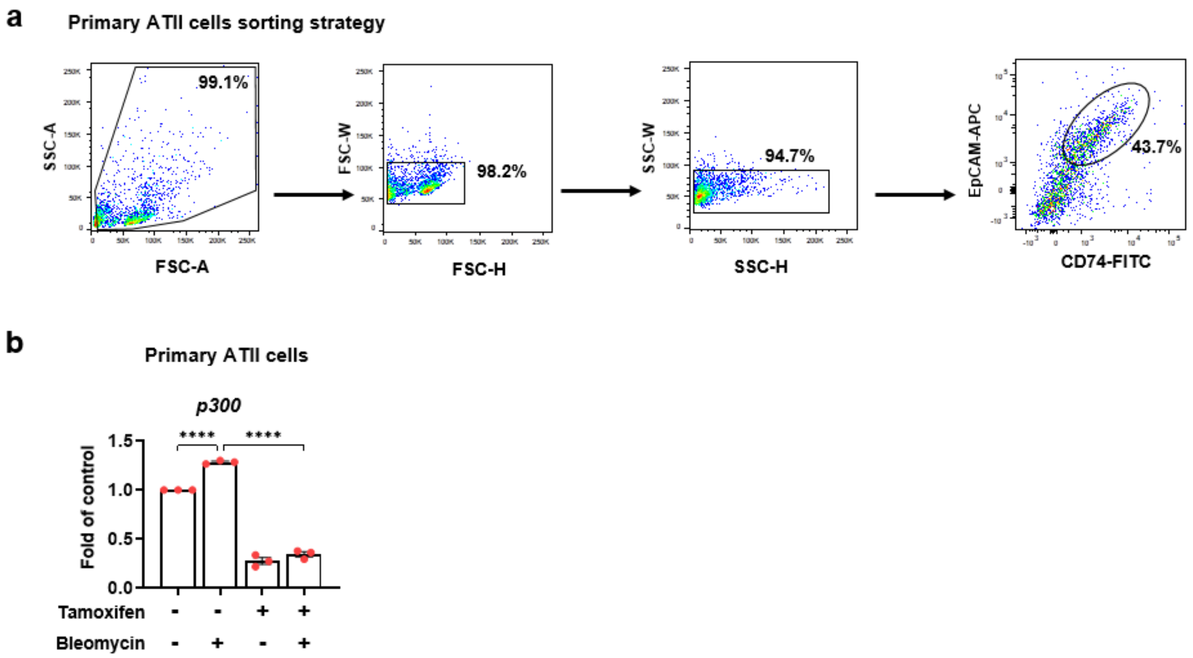

Supplementary Fig. 5: Isolation of primary ATII cells from the lungs of *Spc-p300*$^{f/f}$ mice with or without BLM treatment.

a Gating and sorting strategies for the isolation of primary ATII cells (CD74^{+}EpCAM^{+}) are shown. Cells were stained with CD74-APC and EpCAM-FITC antibodies, and then selected based on forward scatter (FSC) and side scatter (SSC; gated cell population). Aggregates and doublets are excluded using side and forward scatter areas versus their respective width parameters. Next, gating on ATII cells was used for sorting of double-positive population. **b** After vehicle or tamoxifen treatment, isolated ATII cells from Spc-p300$^{f/f}$ mice treated with or without BLM injection were used to perform qRT-PCR analysis. Error bars represent the mean ± s.e.m. of n = 3 mice per group. One-way ANOVA with Tukey's test. ****P < 0.0001.

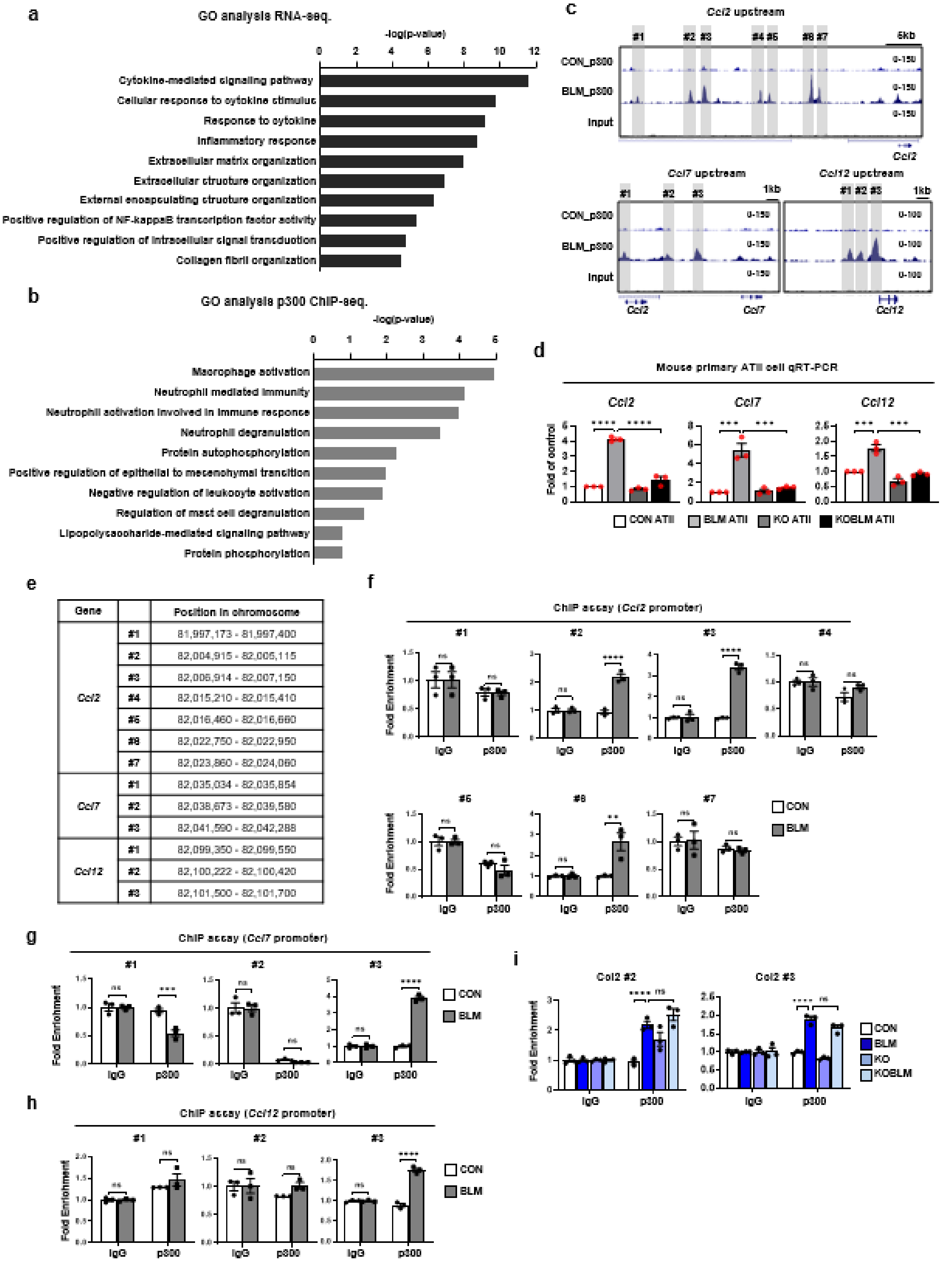

Supplementary Fig. 6: Gene Ontology analysis of RNA-seq and ChIP-seq and identification of p300 binding sites (BEs) in *Ccl2*, *Ccl7*, and *Ccl12* genes.

a, b Gene ontology analysis of differentially expressed genes (DEGs) in RNA-seq (a) and p300-binding increased genes in ChIP-seq (b) data using EnrichR. The lengths of bars indicate the log 10-transformed *P*-value. **c** UCSC genome browser visualizes the p300 binding sites of *Ccl2*, *Ccl7* and *Ccl12* upstream in the lungs of PBS or BLM-treated mice. **d** Primary ATII cells were isolated from PBS or BLM-treated *Spc-p300*$^{f/f}$ or *Spc-p300*$^{d/d}$ mouse lung. The levels of indicated genes were analyzed by qRT-PCR. $n = 3$. **e** Putative p300 binding sites upstream of *Ccl2*, *Ccl7,* and *Ccl12* genes based on ChIP-seq. The numbers indicate the distances from each gene. **f-h** ChIP assay demonstrated that p300 binding upstream of (f) *Ccl2*, (g) *Ccl7,* and (h) *Ccl12* genes increased in the fibrotic lung compared with the normal lung. Error bars represent the mean ± s.e.m. of $n = 3$ mice per group. **i** Recruitment of p300 to the upstream of *Ccl2* had no differences when *p300* gene was knockout in ATII cells. ChIP assay was performed using the indicated antibodies. DNA samples were analyzed by qPCR ($n = 3$). Statistical analysis was performed with two-tailed Mann-Whitney test. Error bars represent mean ± s.e.m. ns, not significant, $^{**}P < 0.01$, $^{***}P < 0.001$, $^{****}P < 0.0001$.

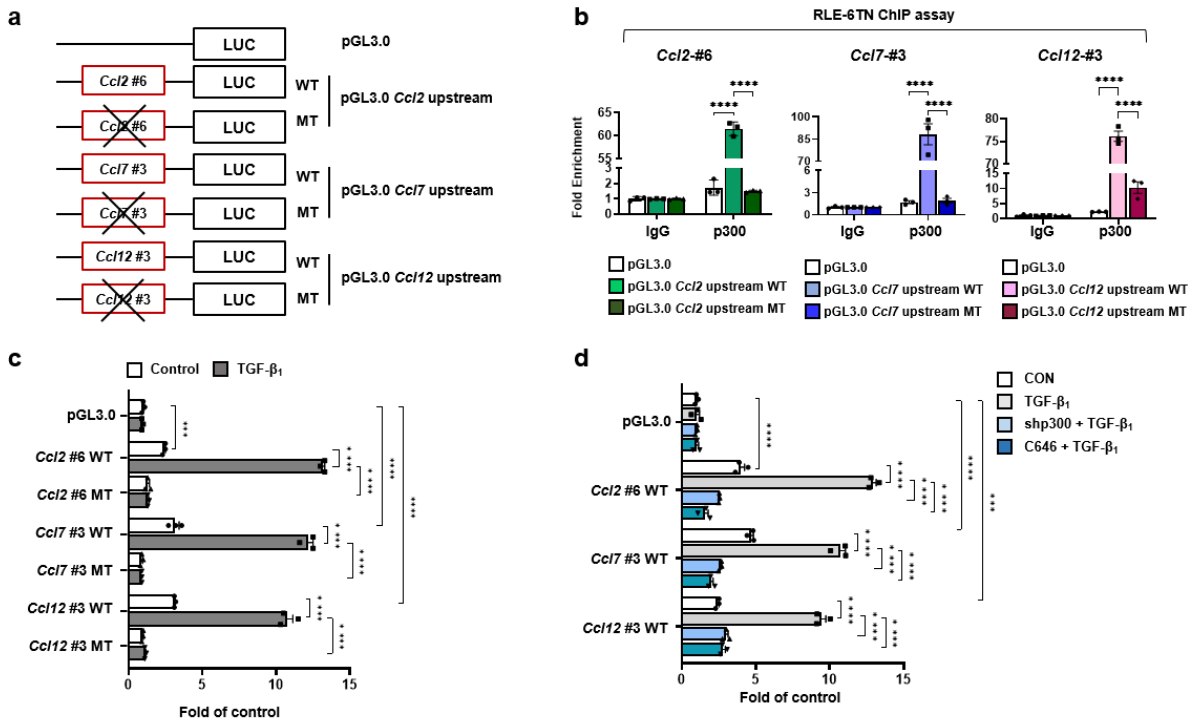

Supplementary Fig. 7: p300-dependent transcriptional activities of *Ccl2*, *Ccl7*, and *Ccl12* gene promotors.

a Schematics of each gene promoter plasmid. **b** RLE-6TN cells was transfected with WT or MT plasmid containing p300-BE of respective *Ccl2*, *Ccl7*, or *Ccl12* gene. **c** Luciferase reporter assay was performed using wild-type (WT) or mutant (MT) constructs of the p300-binding sites for chemokine genes with or without TGF-β_1 in RLE-6TN cells. **d** RLE-6TN cells were transfected with indicated plasmids and/or shRNA against p300. C646 treatment was performed at 2 hours before TGF-β_1 treatment. After then, TGF-β_1 was treated with cells and the luciferase activities were measured after 24 hours. One-way ANOVA with Tukey's test. Error bars represent the mean ± s.e.m. of n = 3 mice per group. ns, not significant, ***P < 0.001 and ****P < 0.0001.

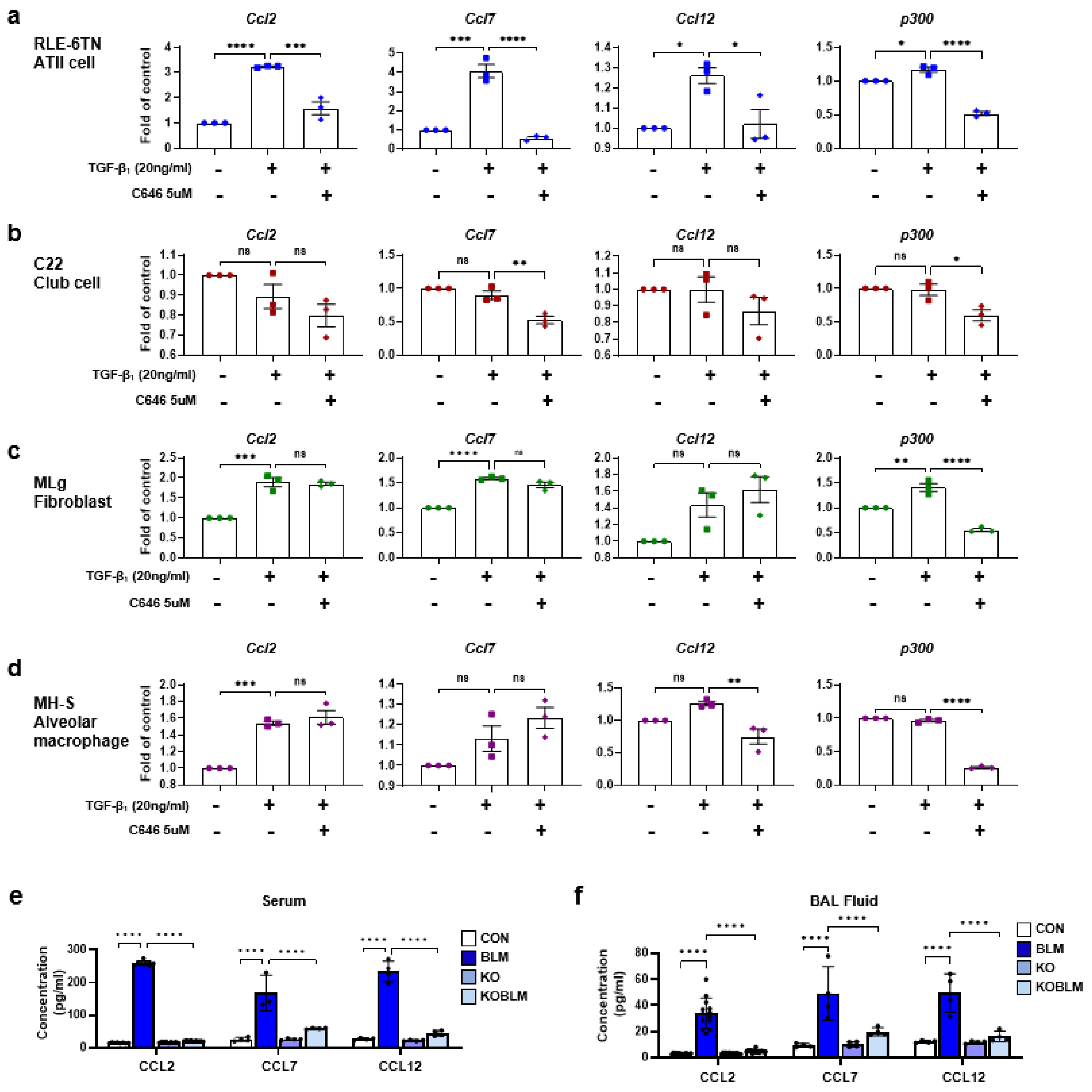

Supplementary Fig. 8: Inhibition of p300 inhibits mRNA and protein expression of *Ccl2, Ccl7*, and *Ccl12* genes in ATII cells.

a-d (a) RLE-6TN ATII cells, (b) C22 club cells, (c) MLg fibroblast cells, and (d) MH-S macrophage cells were treated with C646. C646 treatment was performed 2 hours before TGF-β_1 treatment. After 24 hours of TGF-β_1 treatment, the cells were harvested. Relative expression levels of indicated genes were measured by qRT-PCR. **e, f** Enzyme-linked immunosorbent assay of CCL2, CCL7 and CCL12 protein in serum (e) and BAL fluid (f) of mouse sample. Error bars represent the mean ± s.e.m. of n = 3 mice per group. Statistical analysis was

performed with one-way ANOVA (a-d) or two-way ANOVA (e, f) with Tukey's test. ns, not significant, *$P < 0.05$, **$P < 0.01$, ***$P < 0.001$ and ****$P < 0.0001$.

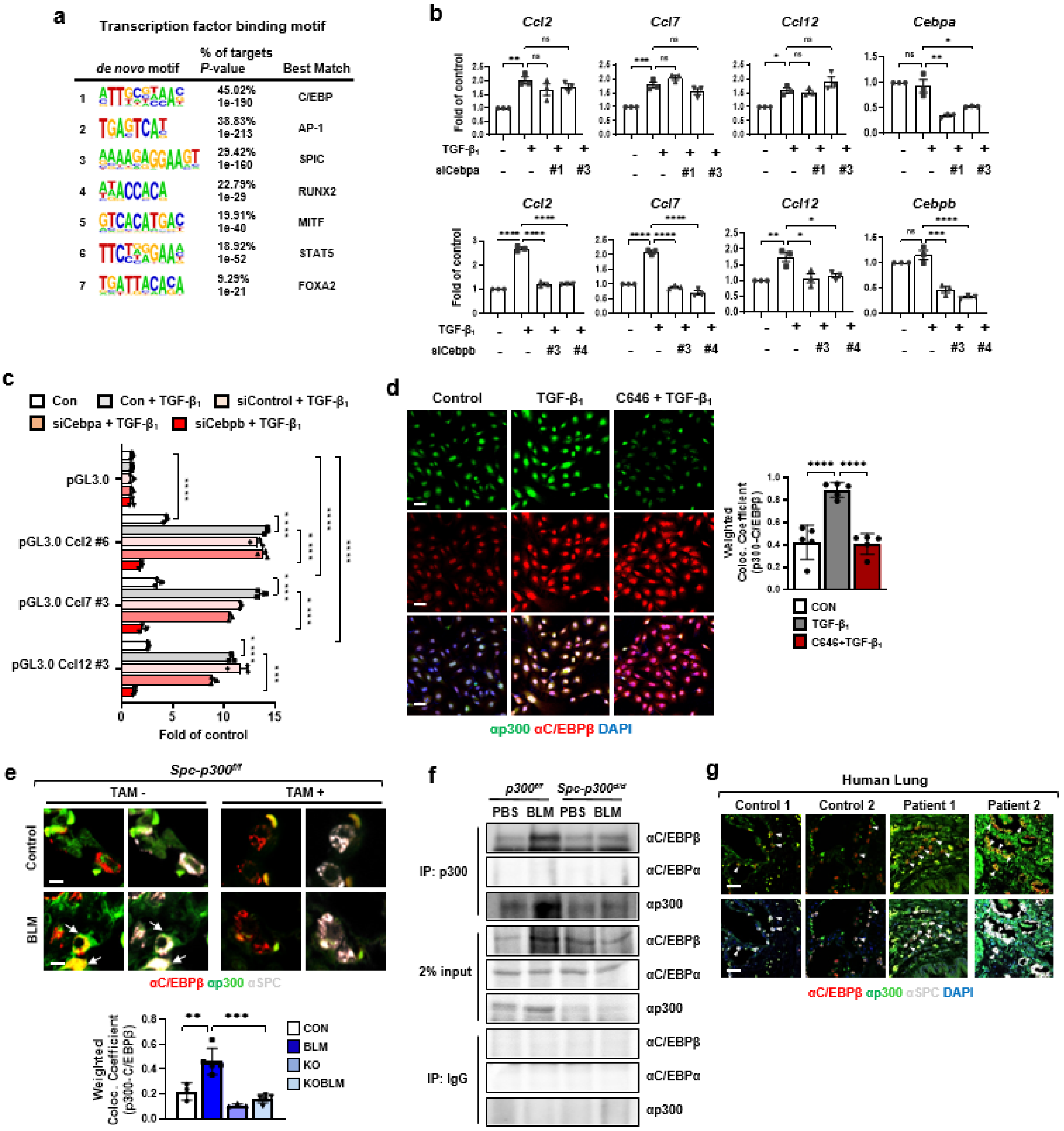

Supplementary Fig. 9: C/EBPβ but not C/EBPα interacts with p300 to mediate the transcriptional activation of *Ccl2*, *Ccl7*, and *Ccl12* genes in ATII cells.

a De novo motif analysis of p300 binding sites using HOMER. **b** Relative expression levels of chemokine genes were determined by qRT-PCR when *Cebpa* or *Cebpb* was knocked down. **c** Luciferase reporter assay was performed using p300-binding site constructs for chemokine genes when *Cebpa* or *Cebpb* was knocked down. **d** Colocalization of p300 (green) and C/EBPβ (red) in RLE-6TN ATII cells by IF staining. Scale bars, 20 μm. Weighted co-localized

coefficient was calculated using ZEN 3.0 software. *n* = 3 per group. **e** Representative immunofluorescence images obtained using confocal microscopy of ATII cells of lung fibrosis mouse model from *Spc-p300*$^{f/f}$ or *Spc-p300*$^{d/d}$ stained with C/EBPβ (red), p300 (green), SPC (grey). Arrows indicate co-localized regions. *n* =3 for group. Scale bars = 5 μm. Weighted co-localized coefficient was calculated using ZEN 3.0 software. **f** Immunoprecipitation assay for p300 in PBS- or BLM-treated lung samples from *Spc-p300*$^{f/f}$ and *Spc-p300*$^{d/d}$ mice. **g** Human lung samples were stained with indicated antibodies; healthy individuals (*n* =14), IPF patients (*n* = 44). Arrowheads indicate co-localized region in ATII cells. Scale bar = 50 μm. Error bars represent the mean ± s.e.m. of *n* = 3 mice per group. Statistical analysis was performed with one-way ANOVA with Tukey's test. ns, not significant, *$P < 0.05$, **$P < 0.01$, ***$P < 0.001$ and ****$P < 0.0001$.

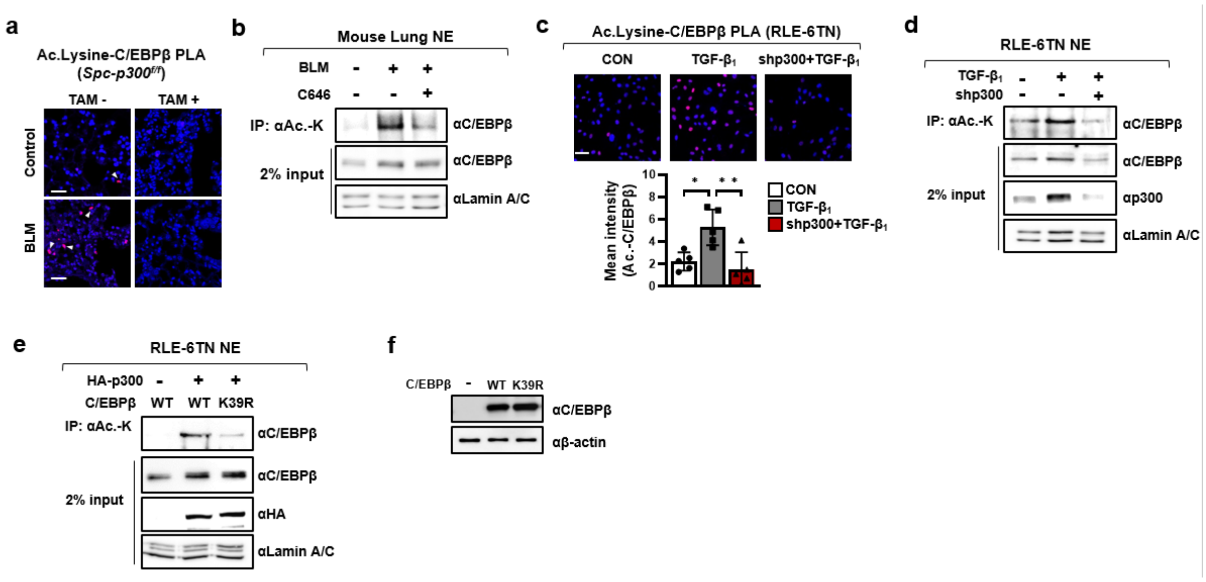

Supplementary Fig. 10: p300 acetylates C/EBPβ to promote TGF-β_1-induced transcriptional activation of chemokine genes in ATII cells.

a Analysis of a proximity ligation assay (PLA) by visualization of the acetylated C/EBPβ protein in *Spc-p300*$^{f/f}$ or *Spc-p300*$^{d/d}$ mice treated with or without BLM. *n* =3 for group. Scale bar = 50 μm. Arrowheads indicate positive signals. **b** Nuclear extract (NE) immunoprecipitation assay for acetylated C/EBPβ was performed in lungs from BLM-treated mice treated with control or C646. **c** RLE-6TN and shp300 RLE-6TN cells were treated with TGF-β_1 for 6 h and analyzed by *in situ* PLA using acetyl-lysine and C/EBPβ antibodies. Scale bars, 20 μm. Mean intensity was quantified using ZEN software. (*n* = 5 per group) **d** Nuclear extracts were immunoprecipitated with acetyl-lysine antibody and immunoblotted with the indicated antibodies. **e** Cells were transfected with the indicated constructs, and immunoprecipitation assay was performed with the indicated antibodies. **f** RLE-6TN cells were transfected with the indicated constructs, and immunoblotting was performed with the indicated antibodies. Error bars represent the mean ± s.e.m. *n* = 3 mice per group. One-way ANOVA with Tukey's test. *$P < 0.05$, **$P < 0.01$.

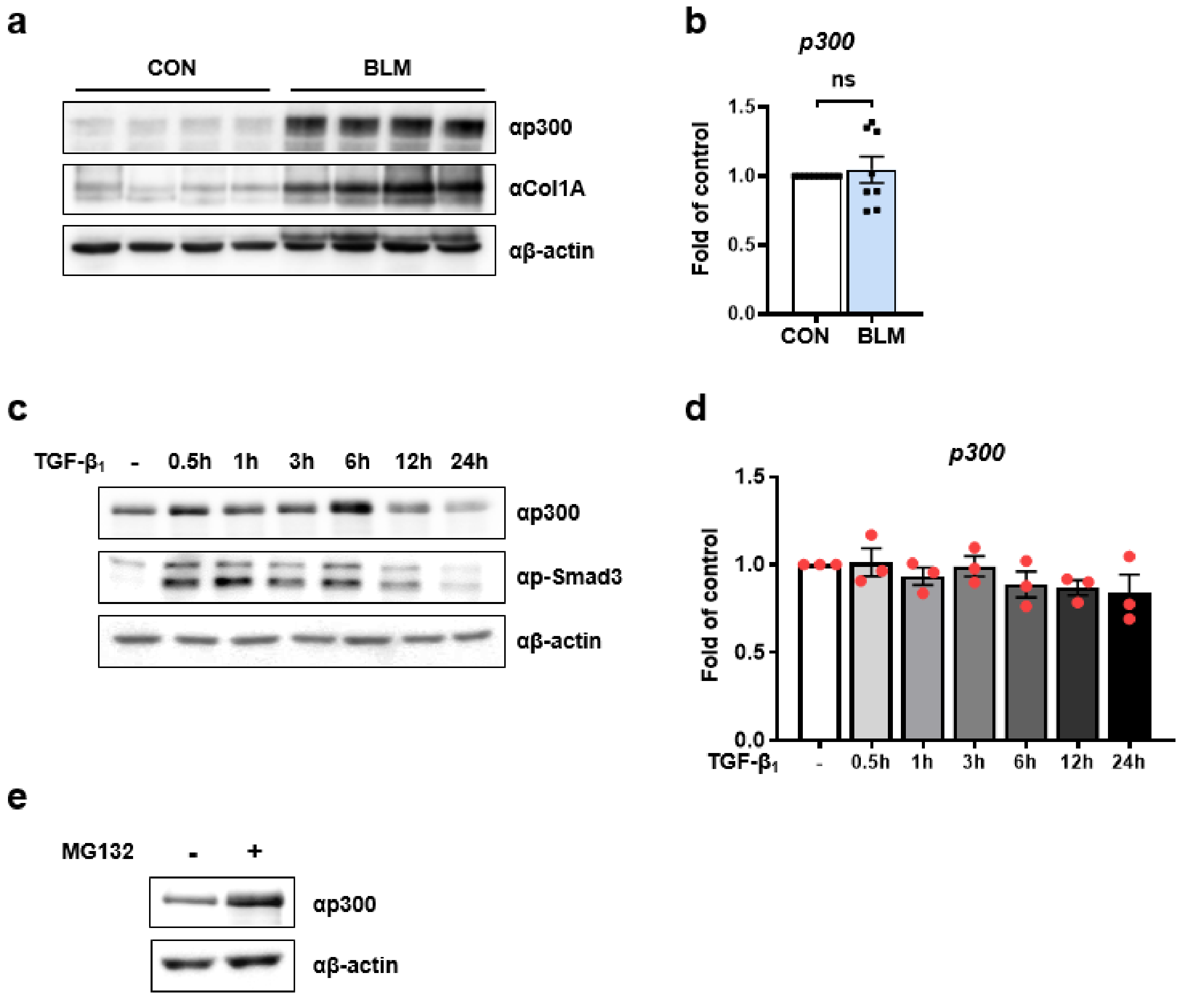

Supplementary Fig. 11: p300 protein but not mRNA levels are increased in lung samples of lung fibrosis mouse model and TGF-β$_1$-treated RLE-6TN ATII cell lines.

a Immunoblotting of lung lysates of lung fibrosis mouse model and control mice. **b** qRT-PCR of lung RNA of lung fibrosis mouse model and control mice. (n = 6 per group). **c** After 2 hours of serum starvation, RLE-6TN was treated TGF-β$_1$ (20 ng/ml) and harvested at indicated time for immunoblotting assay. **d** Relative expression levels of *p300* genes were measured by qRT-PCR. TGF-β$_1$-treated samples were not statistically significant relative to control. **e** RLE-6TN ATII cells were treated with MG132 (10 μM) for 6 h. The expression levels of p300 were detected by immunoblotting assay. Error bars represent the mean ± s.e.m. of n = 3 mice per

group. One-way ANOVA with Tukey's test. ns, not significant, *$P < 0.05$, **$P < 0.01$, ***$P < 0.001$ and ****$P < 0.0001$.

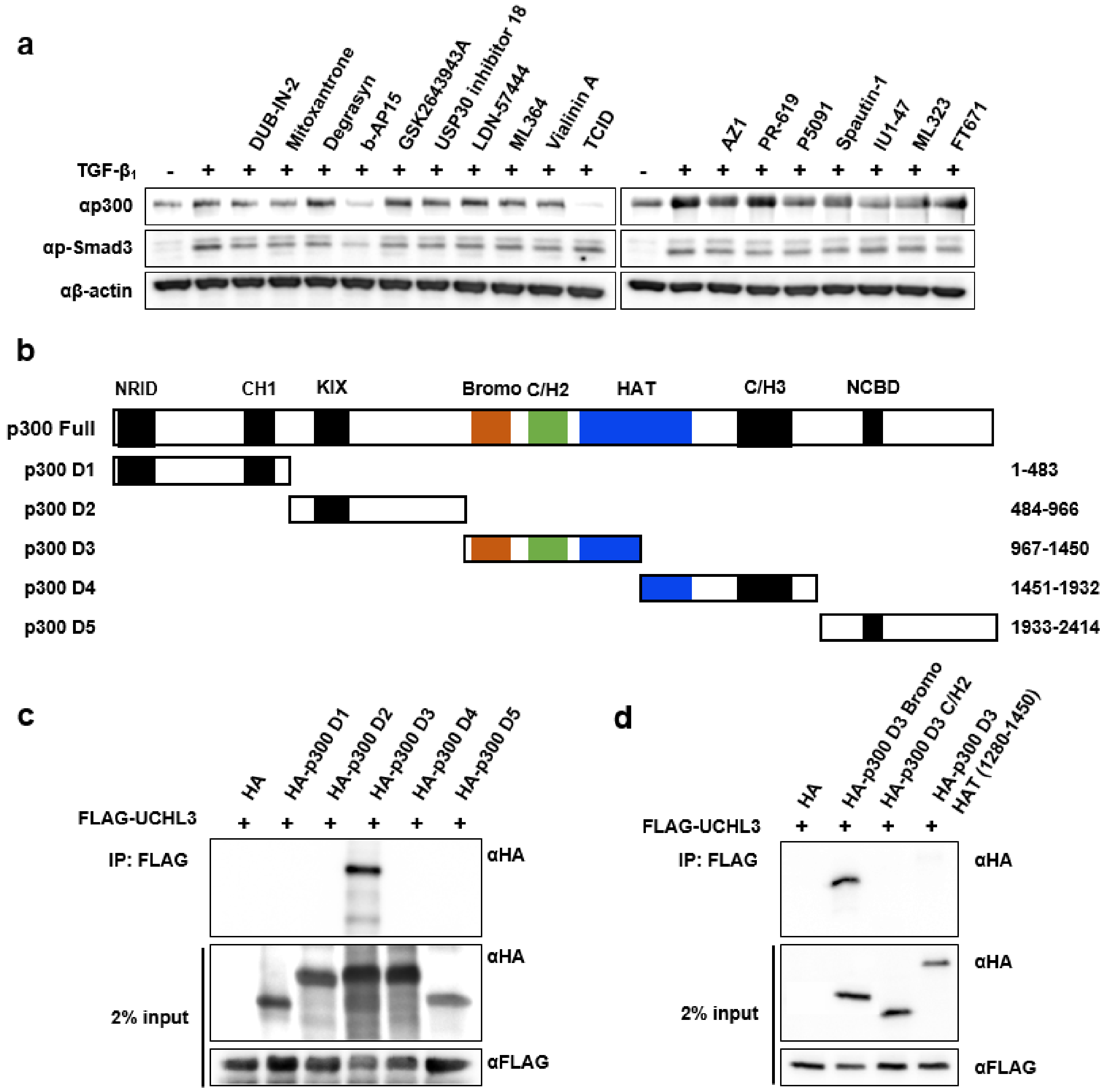

Supplementary Fig. 12: Interaction between p300 and UCHL3 through specific domains.

a Immunoblotting analysis to evaluate inhibition of p300 protein level by using DUB inhibitors. **b** Illustration of p300 domains and the deletion mutants. NRID (nuclear receptor interaction domain), C/H1, C/H2, and C/H3 (cysteine-histidin-rich (CH) region 1, 2, and 3), KIX (kinase inducible domain of CREB interacting domain), Bromo (bromodomain), HAT (histone acetyltransferase), NCBD (nuclear co-activator-binding domain). **c** In vitro transcription/translation and co-immunoprecipitation of UCHL3 and p300 domain plasmids. The samples were immunoprecipitated with anti-FLAG or anti-MYC. **d** In vitro

transcription/translation and co-immunoprecipitation of UCHL3 and each domain of p300 D3 plasmid.

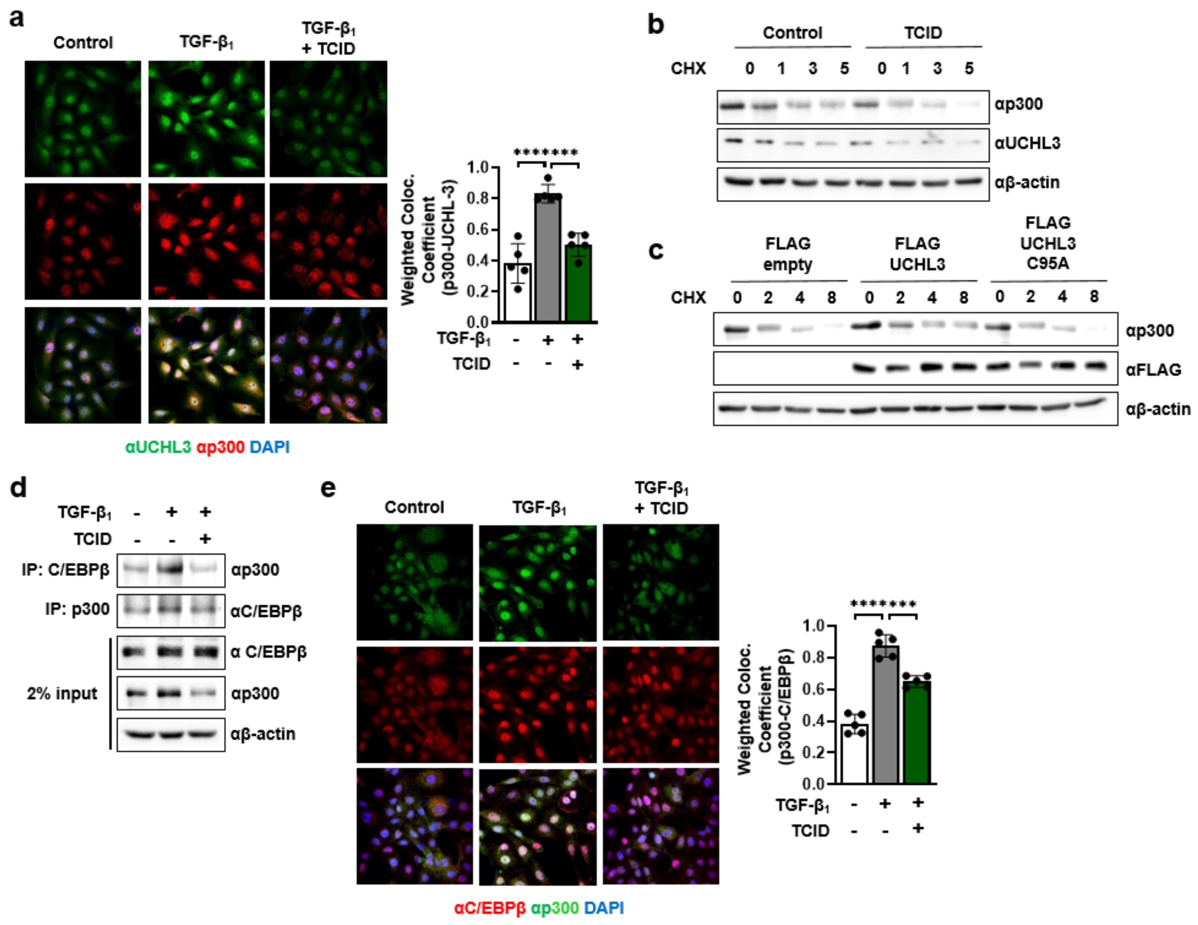

Supplementary Fig. 13: TCID treatment in RLE-6TN ATII cell line stabilizes p300 protein levels and affects CCL2/7/12 chemokine expression and interactions between p300 and C/EBPβ.

a Colocalization of p300 (red) and UCHL3 (green) in RLE-6TN ATII cells by IF staining. Scale bars, 20 µm. Weighted co-localized coefficient was calculated using ZEN 3.0 software. *n* = 3 per group. **b, c** Western blot analysis of indicated proteins in RLE-6TN cell lysates. β-actin was used as loading control. **d** Lysates of RLE-6TN cells were immunoprecipitated with p300 and C/EBPβ antibody and immunoblotted with the indicated antibodies. **e** Colocalization of p300 (green) and C/EBPβ (red) in RLE-6TN ATII cells by IF staining. Scale bars, 20 µm. Weighted co-localized coefficient was calculated using ZEN 3.0 software. *n* = 3 per group. Error bars represent the mean ± s.e.m. of *n* = 3 mice per group. One-way ANOVA with Tukey's test. ns, not significant, *$P < 0.05$, **$P < 0.01$, ***$P < 0.001$ and ****$P < 0.0001$.

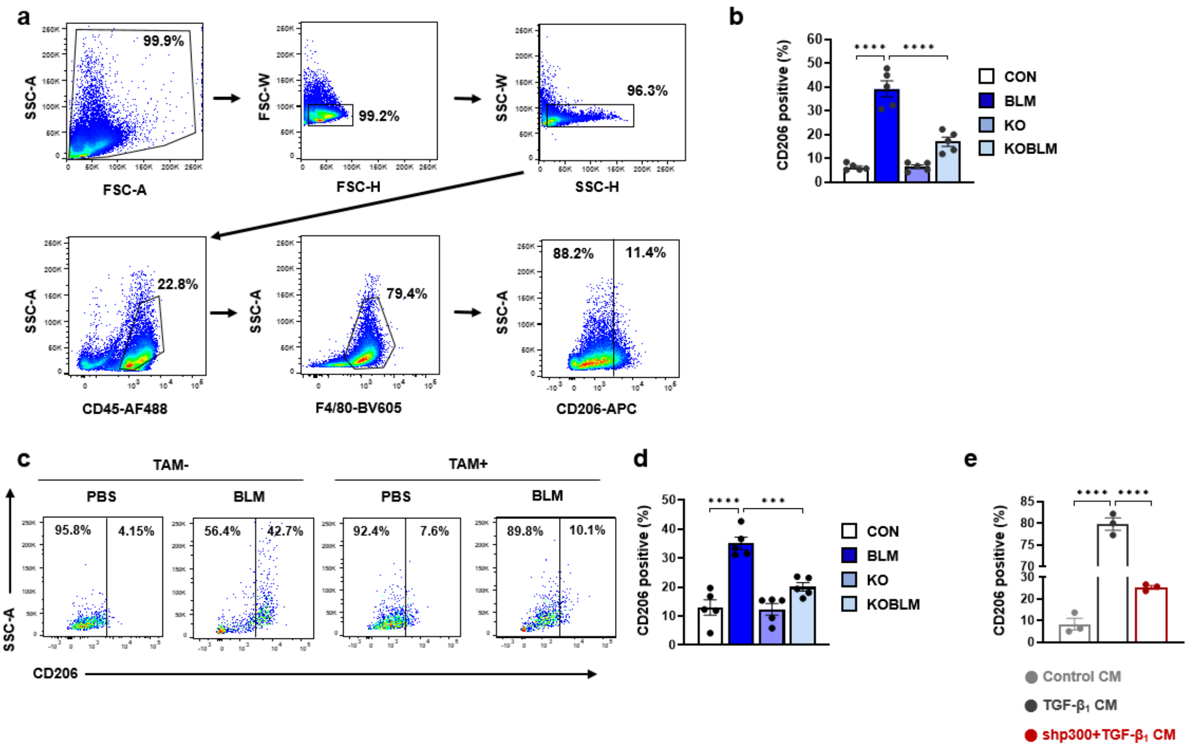

Supplementary Fig. 14: Flow cytometry analysis for M2 macrophages in the lungs of *Spc-p300*$^{f/f}$ mice.

a Gating strategy of macrophage polarization in lung samples using CD45, F4/80 and CD206 antibodies. Sorted cells were selected on the basis of FSC and SSC characteristic. Aggregates and doublets are excluded using side and forward scatter areas versus their respective width parameters. Single cells were analyzed for CD45 positivity, and $CD45^+$ cells were then examined for F4/80 positive expression. The $CD45^+F4/80^+$ cells were analyzed CD206-positive, M2 macrophage cells. **b** Quantification of the M2 macrophages from mouse lungs. n =5 per group. **c** Cells in BAL fluid were analyzed by flow cytometry BLM injection. **d** The graph indicates the percentage of M2 positive macrophage in BAL cells of the groups. **e** Quantification of CD206-positive cells in MH-S macrophages cocultured with or without CM from control or p300 knockdown RLE-6TN cells. n = 5 per group. Error bars represent mean ± s.e.m. ***$P < 0.001$ and ****$P < 0.0001$, one-way ANOVA followed by Tukey's test.

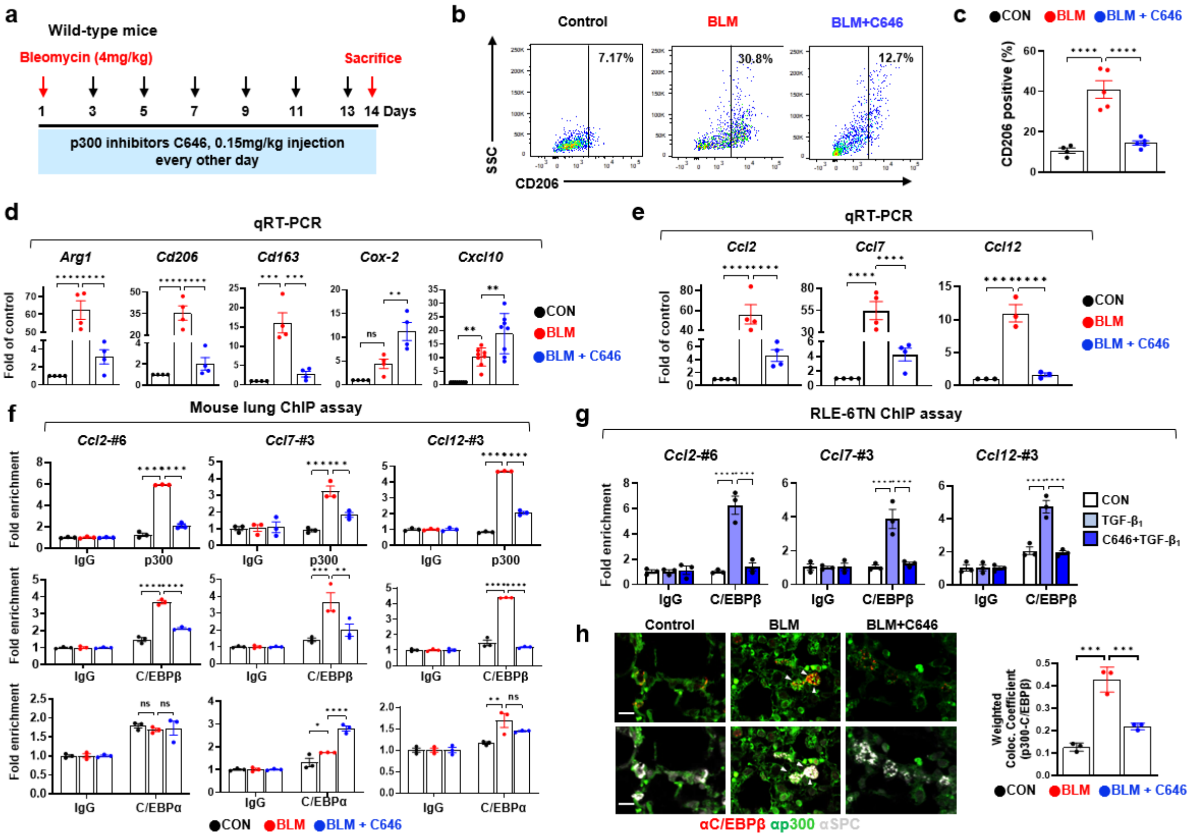

Supplementary Fig. 15: Inhibition of p300 activity prevents the progression of lung fibrosis.

a Flow chart for the C646 injection mouse experiment. Animals were divided into three groups: control, only BLM treatment, or BLM treatment with C646 injection. C646 was administered intraperitoneally every other day for 2 weeks after BLM treatment. **b** Flow cytometry analysis for M2 macrophages in BAL fluid to examine the effects of C646 injection. **c** The graph indicates the percentage of M2 macrophage in BAL cells of the groups. n = 4-5 mice per group. **d** Relative *Arg1*, *Cd206*, *Cd163*, *Cox-2, and Cxcl10* mRNA levels in C646-injected mouse lung samples. **e** Relative expression of Ccl2, Ccl7, and Ccl12 genes in the lungs of C646-injected mice. **f** ChIP assay for p300, C/EBPβ, and C/EBPα binding with the p300-BE of *Ccl2*, *Ccl7*, and *Ccl12* relative to input DNA in lung samples from C646-injected mice. n =3 each. **g** ChIP assay for C/EBPβ was conducted in C646-treated RLE-6TN ATII cells. n = 3. **h** Colocalization of p300 (green), C/EBPβ (red), and SPC (grey) by IF staining in indicated mouse lung samples.

Scale bar, 50 μm. $n = 3$. The graph indicates weighted co-localized coefficiency. Error bars represent mean ± s.e.m. ns, not significant, $^{**}P < 0.01$, $^{***}P < 0.001$ and $^{****}P < 0.0001$, one-way ANOVA followed by Tukey's test.

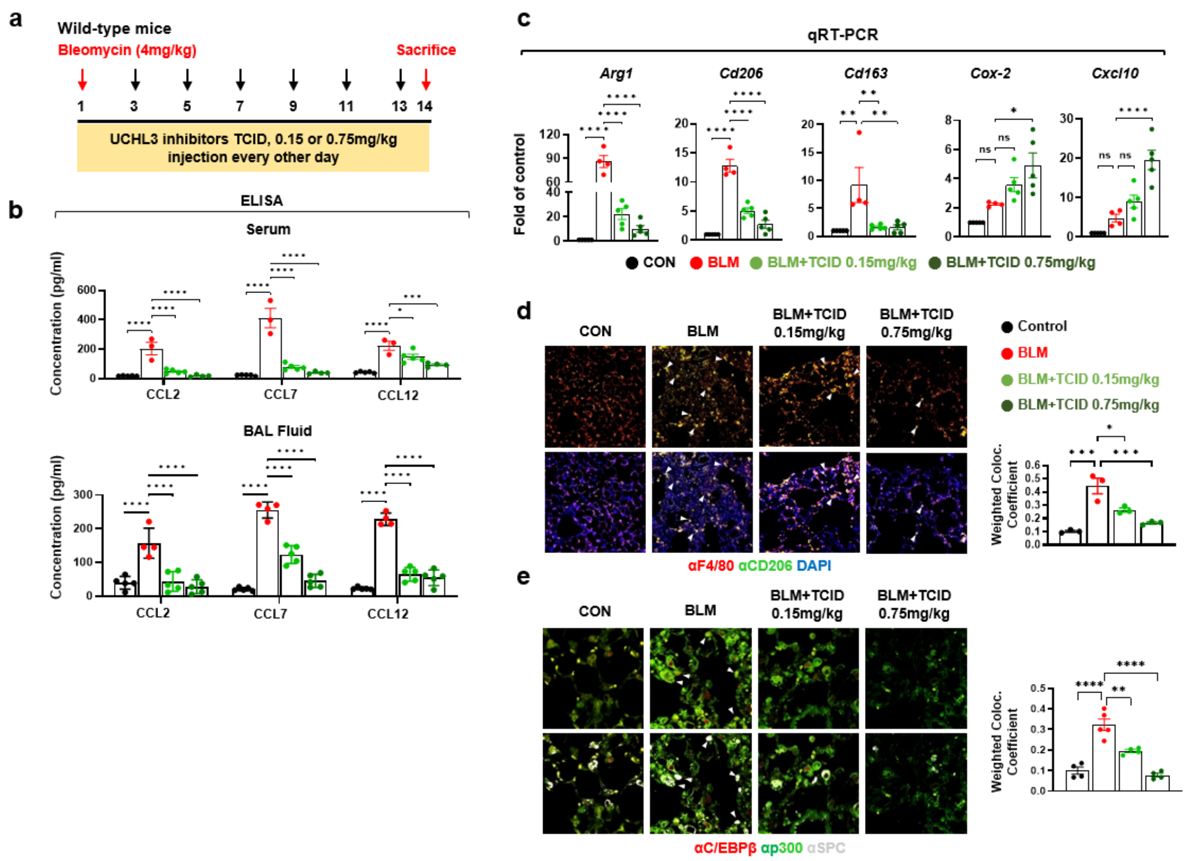

Figure S16. Treatment of TCID alleviates the progression of bleomycin-induced lung fibrosis.

a Flow chart for the TCID injection mouse experiment. Wild-type mice were divided into four groups: control, only BLM treatment, BLM treatment with 0.15 mg/kg TCID, and BLM treatment with 0.75 mg/kg TCID. TCID was administered intraperioneally every other day for 2 weeks after BLM treatment. **b** ELISA analysis of CCL2, CCL7, and CCL12 protein in serum and BAL fluid from control or TCID-treated mice. **c** Relative *Arg1*, *Cd206*, *Cd163*, *Cox-2*, and *Cxcl10* mRNA levels in TCID-injected mouse lung samples. **d** IF analysis of CD206 (green) and F4/80 (red) with DAPI (blue) in the mouse lungs. Arrow heads indicated co-localized cells Scale bar, 50 μm. *n* = 3. **e** Colocalization of p300 (red), UCHL3 (green) in Club cells by IF staining in indicated mouse lung samples Scale bar, 50 μm. *n* = 3. The graph indicates weighted

co-localized coefficiency. Error bars represent mean ± s.e.m. ns, not significant, $^{**}P < 0.01$, $^{***}P < 0.001$ and $^{****}P < 0.0001$, one-way ANOVA followed by Tukey's test.

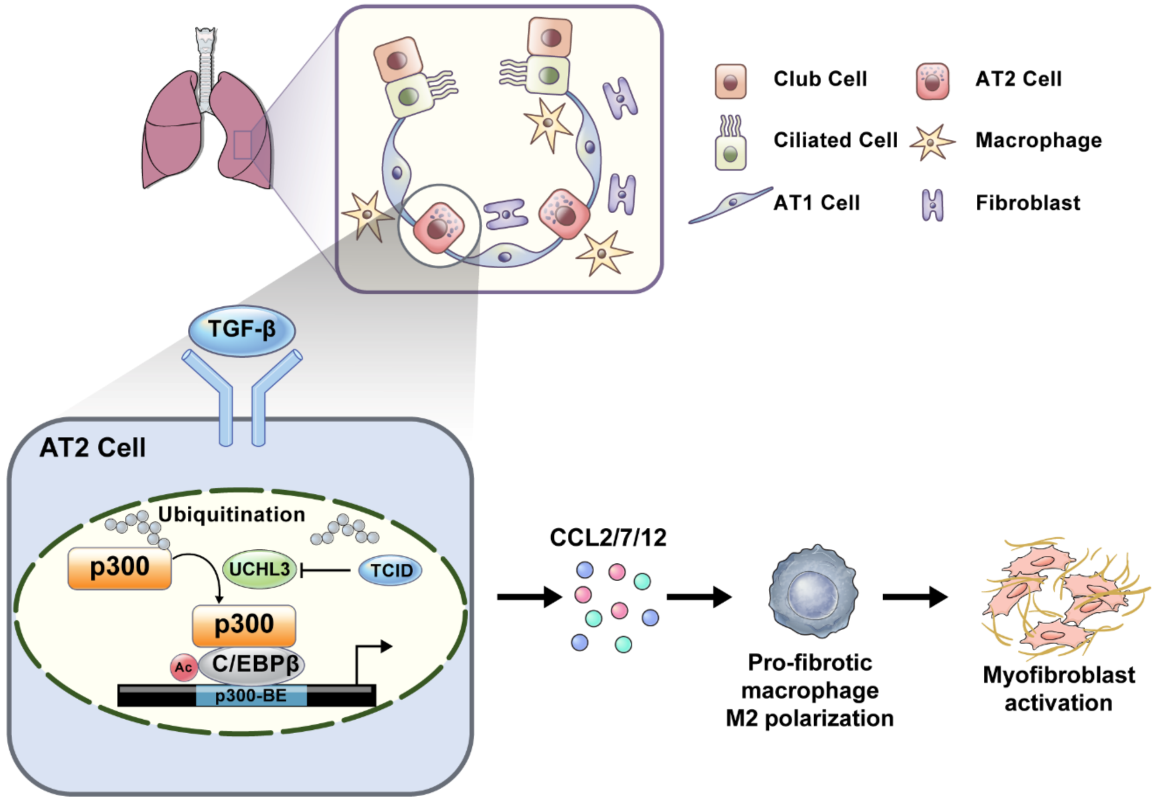

Supplementary Fig. 17: Schematic model of study findings.

Upon external stimuli in lung ATII cells, UCHL3 stabilzes p300 and p300 mediates the TGF-β-induced transcriptional activation of *Ccl2*, *Ccl7*, and *Ccl12 chemokine genes* through the cooperative action of C/EBPβ. The activation of chemokines induces macrophage polarization, which leads to the progression of lung fibrosis.
